# Nicotine Dependence and Quit Self-Confidence in a Smoking Cessation Program Using a Group-Based Digital Peer-Supported App and Cigarette Consumption–Adjusted Nicotine Aids Among Japanese Workers: Retrospective Cohort Study

**DOI:** 10.2196/84792

**Published:** 2026-05-19

**Authors:** Shota Yoshihara, Kayoko Takahashi, Chiaki Uemura, Shin Murakami, Satoru Amano, Daichi Harada, Ying Jiang, Hiroshi Yamato

**Affiliations:** 1 Department of Rehabilitation Sciences, Graduate School of Medical Sciences, Kitasato University, 1-15-1, Kitasato, Sagamihara, Kanagawa, 252-0373, Japan, 81 0427889694; 2 A10 Lab Inc, Chuo-ku, Tokyo, Japan; 3 School of Allied Health Science, Kitasato University, Sagamihara, Kanagawa, Japan; 4 Department of Health Development, Institute of Industrial Ecological Sciences, University of Occupational and Environmental Health, Japan, Kitakyushu, Fukuoka, Japan

**Keywords:** smoking cessation, nicotine dependence, self-confidence, heated tobacco products, digital health, workplace health promotion

## Abstract

**Background:**

Completion rates for smoking cessation treatments under Japan’s national health insurance system remain suboptimal. A workplace cessation program, combining nicotine gum or patches with a group-based digital peer-supported app, has reported high cessation success rates. Although nicotine dependence is generally associated with lower cessation success, and self-confidence is generally associated with higher success, these associations may differ by tobacco product type. Evidence on these relationships in app-based cessation programs remains limited.

**Objective:**

This study aimed to examine the independent and combined associations of nicotine dependence and self-confidence in quitting with smoking cessation success among cigarette-only smokers, heated tobacco product–only users, and dual users.

**Methods:**

This retrospective cohort study used data from a workplace cessation program in Japan. Participants were eligible if they were employed, owned a smartphone, and self-enrolled in the program. Recruitment was conducted through workplace promotion and individual outreach, primarily via email from companies. The program combined a digital peer-support app with nicotine gum or patches, adjusted according to cigarette consumption. The app included anonymous peer-support group chats of up to 5 participants, where participants shared progress, photos, and comments. Nicotine dependence was assessed by the time to the first cigarette after waking (high: ≤30 min; low: >30 min). Self-confidence for quitting was rated on a 0‐10 scale and dichotomized at the median. A 4-level variable combined nicotine dependence and self-confidence for quitting. Logistic regression analyses were conducted by tobacco product type, and odds ratios (ORs) and 95% CIs were estimated.

**Results:**

A total of 2143 participants were included in the analysis. Their mean age was 46.5 (SD 10.9) years, and approximately 90% were men. Overall cessation success was 53.8% (1152/2143). Participants with high self-confidence had a higher cessation success rate than those with low self-confidence (529/834, 63.4% vs 623/1309, 47.6%), with an OR of 1.81 (95% CI 1.55‐2.12). Nicotine dependence was significantly associated with cessation success only among cigarette-only smokers; those with low nicotine dependence had a higher OR than those with high dependence (OR 1.59, 95% CI 1.07‐2.37). Across all tobacco product types, the subgroup with low nicotine dependence and high self-confidence showed the highest cessation success, followed by the subgroup with high nicotine dependence but high self-confidence.

**Conclusions:**

By distinguishing cigarette-only smokers, heated tobacco product–only users, and dual users in a workplace cessation program, this study provides novel, product-specific evidence regarding nicotine dependence and self-confidence related to cessation success. This study extends prior research beyond cigarette-only smokers by examining tobacco user groups and suggests that self-confidence may predict cessation across tobacco product types, whereas the role of nicotine dependence may be product-specific. These findings may inform tailored, scalable smoking-cessation support in workplaces; however, they should be interpreted with caution because cessation outcomes were self-reported.

## Introduction

Smoking is the leading cause of mortality from noncommunicable diseases in Japan and contributes to the incidence of ischemic heart disease, stroke, chronic obstructive pulmonary disease, and cancer [1,2]. As the societal burden of smoking increases, smoking cessation has become a global imperative [3]. Despite global and national efforts to strengthen tobacco control policies, the desire to quit smoking among current smokers in Japan remains low at 20.7% [4]. Recent national survey data indicate that conventional cigarette-only smokers, heated tobacco product (HTP)-only users, and dual users account for 60.5%, 29.2%, and 9.2% of current male smokers and 56.2%, 35.3%, and 7% of current female smokers, respectively [4].

In Japan, a standard outpatient smoking cessation program is available for smokers with nicotine dependence, including HTP users [5]. This program includes a 12-week course of nicotine replacement therapy using nicotine patches, delivered over 5 sessions, along with counseling provided by physicians either in person or via telemedicine. However, the completion rate of this smoking cessation program remains low.

Several systematic reviews have reported that digital interventions, such as smartphone apps, can aid smoking cessation [6,7]. Moreover, a meta-analysis indicated that group-based interventions incorporating peers enhanced smoking cessation success rates [8]. Our previous study revealed a significantly higher cessation success rate among current smokers using a digital peer-supported app together with nicotine aids in the workplace (59.2%) than among those using only nicotine aids (38.7%) [9]. Furthermore, the cessation success rate was significantly higher among employed smokers using only HTPs (63.3%) than among those using only cigarettes (52.8%) [10].

Multiple factors identified by previous studies as being associated with smoking cessation include age [11,12], educational level [12-14], socioeconomic status and occupation [15], marital or cohabitation status [12,13,15], number of friends who smoke [14], housing conditions [11], and social or family support [13,15]. Among these, nicotine dependence has been consistently recognized as an important factor in smoking cessation [11,16-20]. Additionally, self-confidence for quitting, defined as a smoker’s confidence in their ability to quit, is considered a key psychological factor for smoking cessation. A meta-analysis of 54 prospective studies showed that individuals who remained abstinent at follow-up had higher self-confidence for quitting, such as self-abstinence self-efficacy, than those who continued smoking, and that greater self-efficacy was associated with an increased likelihood of cessation success [21]. Moreover, previous reports have suggested a negative association between self-confidence for quitting and nicotine dependence [22,23].

Nonetheless, most previous studies have focused solely on cigarette smokers, and little is known about whether nicotine dependence and self-confidence for quitting are associated with smoking cessation among HTP or dual users, or whether these associations vary by tobacco product type. This gap in the literature is important because studies have increasingly suggested that smoking cessation success may differ by tobacco product type, indicating the need for product-specific evaluation of factors associated with cessation [24-26]. HTPs are marketed in over 40 countries [27], with Japan being the largest market [28]. HTP users heat tobacco to generate aerosols (vapors) rather than smoke [29] and are marketed as substitutes for cigarettes [30]. Although HTPs emit lower concentrations of combustion-related toxicants, the aerosols produced by HTPs still contain several harmful constituents, including those related to additives such as glycerol [31]. Moreover, the proportion of smokers intending to quit in Japan has declined since 2014, in parallel with increasing HTP use [4], suggesting that the promotion of cessation among HTP users represents a public health issue [32].

To address these gaps, in the present study, we investigated the following research questions in a company-implemented smoking cessation program combining a digital peer-support app with nicotine gum or patches:

- Are nicotine dependence and self-confidence for quitting independently associated with smoking cessation success?
- Do these associations vary by tobacco product type (cigarette-only smokers, HTP-only users, and dual users)?
- What is the joint association of nicotine dependence and self-confidence for quitting with smoking cessation success across these tobacco product groups?

## Methods

### Study Design

This retrospective cohort study evaluated differences in smoking cessation success rates according to nicotine dependence and self-confidence in quitting among users of a group-based digital peer-supported app and nicotine aids (nicotine patches or gum). The study was conducted among current smokers working at 38 companies from May 2024 to March 2025.

### Sampling Procedures

Participants were recruited for a company-implemented, workplace-based smoking cessation program using a convenience sampling approach based on self-selection through individual outreach and workplace promotion over approximately 3 weeks, beginning about 2 weeks before the program started. Participants were invited primarily via email from their companies and union officials associated with their health insurance societies. In addition, posters and other promotional materials were displayed on company bulletin boards to raise awareness. Participants enrolled in the smoking cessation program by independently submitting the provided application form (eg, Google Forms).

### Participant Inclusion and Exclusion Criteria

Current smokers enrolled in the smoking cessation program who used the digital peer-supported app were included in this retrospective analysis. Eligible participants were individuals who owned either an iOS or Android smartphone and were enrolled in their company’s corporate health insurance program. Smokers were defined as participants who reported smoking at least 1 cigarette per day, including novel tobacco products such as HTPs and e-cigarettes. Participants who never used the digital peer-supported app, or nicotine gum or patches, as well as those with missing outcome data, were excluded from the primary analysis.

### Smoking Cessation Program

**Figure 1.**
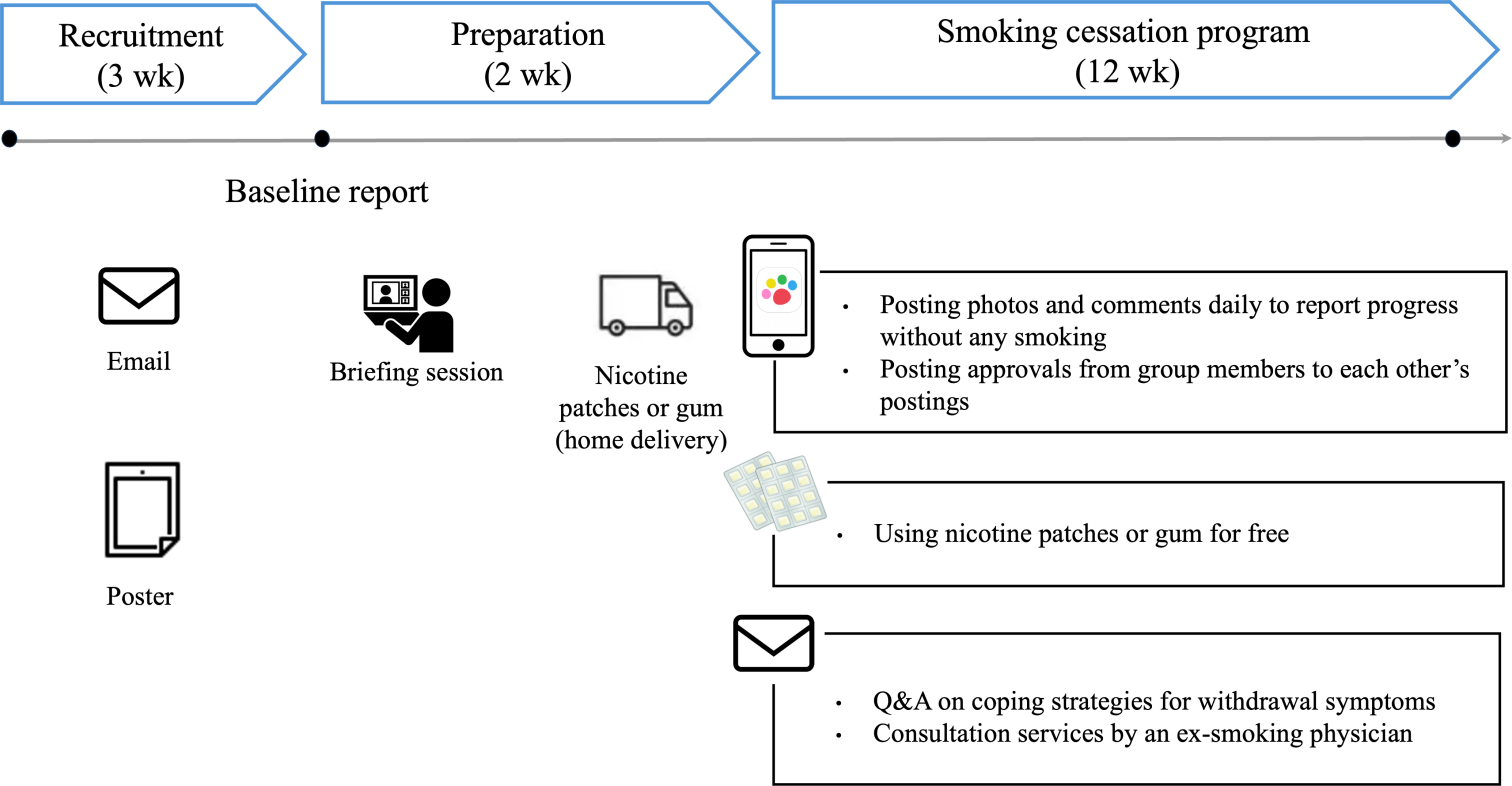
Timeline of study procedures.

Our smoking cessation program, which lasted for 12 weeks, was conducted remotely by each company (Figure 1). In the preprogram phase, the program’s structure was explained during a real-time web-based briefing session, which included educational content led by a physician who had quit smoking. The session covered topics such as the health effects of smoking, the benefits of quitting smoking, coping with withdrawal, creating a smoke-free environment, and adopting alternative behaviors. The digital peer-supported app was also introduced, and the proper use of nicotine patches and gum was demonstrated. For those who were unable to attend a live session, the same content was made available in an e-learning course.

As summarized in Table 1, nicotine patches or gum were provided according to the participants’ daily cigarette consumption (1‐4, 5‐10, or ≥11 cigarettes per day), workplace environment, and preference regarding gum use.

After the web-based briefing session, the participants received complimentary nicotine patches or gum, which were available over the counter, and began their smoking cessation efforts using the digital peer-supported app. Additional support, such as “Q&A on Coping Strategies for Withdrawal Symptoms,” was provided through a dedicated email inquiry service, and an ex-smoking physician specializing in smoking cessation offered consultation services.

**Table 1.** Provision patterns of nicotine gum or patches.

Number of cigarettes/day	Gum allowed at the workplace	Gum not allowed at the workplace
≥11	Pattern A: 7 sheets of 20 cm² patches +96 pieces of gum	Pattern C: 14 sheets of 20 cm² patches +14 sheets of 10 cm² patches
5‐10	Pattern B: 14 sheets of 10 cm² patches +48 pieces of gum	Pattern D: 20 sheets of 10 cm² patches
1-4	Pattern E^a^: 96 pieces of gum alone	Pattern E^a^: 96 pieces of gum alone

a Based on physician consultation, a 10 cm² patch was considered too strong for individuals who smoke 1‐4 cigarettes/day; hence, the gum-only regimen was applied uniformly to this group, irrespective of workplace gum restrictions.

### Digital Peer-Supported App

A commercially available digital peer-supported app (“Minchalle”; A10 Lab Inc), accessible on both iOS and Android smartphones, was used in this study [9,10,33-36]. This app was originally developed to help users build desirable habits, such as exercising, dieting, and learning English conversations, and it has been downloaded more than 1.6 million times. In this study, the app was adapted to support smoking cessation [9,10].

**Figure 2.**
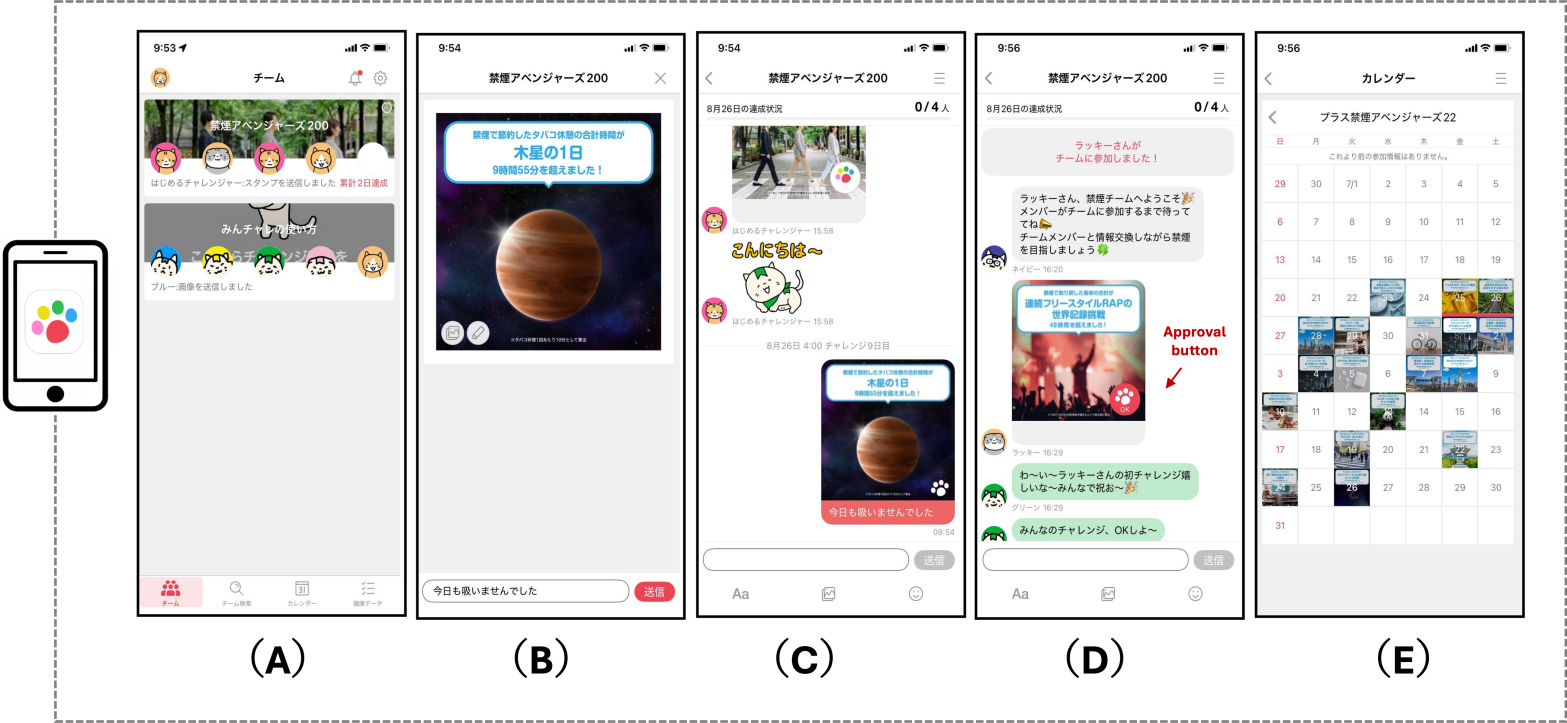
Examples of app screens. (A) Selecting a group consisting of 5 members, (B) uploading a photo of the day with a comment related to abstinence, (C) displaying the content of posts in the group, (D) responding to posts by group members and pressing the approval button, and (E) reviewing the records of abstinence through photos. These functions are designed to support self-monitoring of abstinence, peer interaction through team-based participation and approvals from group members, and progress tracking within the app.

The participants were automatically assigned to teams comprising 5 individuals based on their registration order in the app. This app facilitated group chats among team members sharing a common goal (in this case, smoking cessation) and promoted anonymous interactions by allowing users to post messages and photos about smoking abstinence (Figure 2). All app interactions were designed to be anonymous, and the participants could use the app freely. In addition to self-monitoring abstinence, the app supported peer interaction through team-based participation, daily posting, approvals from group members, and progress tracking.

Within the app, the participants could (1) post photos and comments daily to report their progress without smoking, (2) post approvals from group members on each other’s posts, (3) receive feedback on the team’s collective performance toward its goals, and (4) be automatically removed from the group if they exceeded a specified inactivity period of 15 consecutive days. The participants posted comments or photographs multiple times daily and interacted with others as they wished, although participation was not mandatory. The user interface, including its layout and interactive features, has been described in detail in previous studies [9,10].

### Data Collection Procedures

Baseline participant characteristics were collected using a web-based survey before the start of the program. App engagement data were automatically extracted from the digital peer-supported app database during the 12-week program. Smoking cessation outcomes were collected at the end of the program using a web-based form, where participants reported their last smoking date.

### Participant Characteristics and Baseline Measures

Baseline data, including age (continuous), sex (male or female), and smoking-related characteristics (smoking duration, number of cigarettes smoked per day, number of previous smoking cessation attempts, importance of smoking cessation, and type of cigarettes smoked), were collected via a web-based survey.

Smoking duration (years) was assessed by asking the question, “How many years have you been smoking?*”* The participants were instructed to select one of the following options: 0‐2, 3‐4, 5‐9, 10‐19, 20‐29, or ≥30 years. Smoking duration was determined without distinguishing between tobacco product types. Given that HTPs have been available for less than 10 years, HTP-only users with a smoking duration of more than 10 years were assumed to have previously used combustible cigarettes but were currently using only HTPs. Cigarette consumption was assessed by asking the question, “How many cigarettes do you currently smoke per day?*”* The participants provided continuous responses. The number of previous smoking cessation attempts was recorded as a categorical variable (0, 1, 2, 3, 4, 5, 6, 7, 8, 9, 10, or ≥11), indicating the number of prior quit attempts before the current program. The importance of smoking cessation was assessed with the item, “How important is it to you that you can quit smoking?*”* rated on a self-reported 0‐10 scale.

### App Engagement Measures

The digital peer-supported app log data automatically recorded in the database were obtained to assess the participants’ engagement with the app. Two data indicators were analyzed: (1) posting frequency during the 12-week program and (2) the number of approval button presses from group members. Posting frequency was defined as the total number of posts (including messages, challenge posts, normal photos, and stamps) made by each participant throughout the program. The number of approval button presses from group members in the app was extracted from log data and regarded as a continuous variable.

### Tobacco Product Type

At baseline, cigarette smoking status was assessed by asking the question, “Which type of cigarette do you currently use?*”* The participants selected from multiple options: (1) combustible cigarettes, (2) HTPs (eg, IQOS, Ploom, glo, PULZE, and WEECKE), or (3) e-cigarettes. The use of e-cigarettes was not considered in this study because the prevalence of e-cigarette use is low in Japan [37-39]; moreover, e-cigarettes in Japan do not contain nicotine. Based on their responses, the participants were categorized into cigarette-only smokers, HTP-only users, and dual users [10].

### Measures of Nicotine Dependence and Self-Confidence for Quitting

Nicotine dependence was assessed by asking the question, “How soon after waking do you smoke your first cigarette?” The participants selected from the following options: within 5 minutes, 6 to 30 minutes, 31 to 60 minutes, or after 60 minutes. Based on previous studies and clinical guidelines [40-42], the participants who responded “within 5 minutes” or “6 to 30 minutes” were categorized into the high nicotine dependence group, whereas the participants who answered “31 to 60 minutes” or “after 60 minutes” were assigned to the low nicotine dependence group.

Self-confidence for quitting was assessed with the item, “How confident are you that you can quit smoking?” rated on a self-reported 0‐10 scale [43-45]. Participants were dichotomized at the median into low and high self-confidence groups.

In this study, a composite 4-level variable was created by combining nicotine dependence (low or high) with self-confidence (low or high): (1) low nicotine dependence and low self-confidence, (2) low nicotine dependence and high self-confidence, (3) high nicotine dependence and low self-confidence, and (4) high nicotine dependence and high self-confidence.

### Outcome Measure: Smoking Cessation Success Rates

Smoking cessation success rates were calculated based on the participants’ self-reported abstinence from smoking for at least 4 consecutive weeks. At the end of the 12-week program, the participants submitted the date when they had last smoked via a web-based form. The participants were considered to have successfully quit smoking if the number of days between their self-reported “last smoking date” and the final report date was at least 4 weeks. Those who reported complete abstinence during this period without smoking a single cigarette were classified as “quitters.”

### Sample Size, Power, and Precision

This retrospective cohort study used data from a company-implemented smoking cessation program. No formal sample size calculation was performed a priori, as the sample size was determined by the number of eligible participants available during the study period. Similarly, no formal power analysis was conducted for the same reason. Precision was assessed by reporting odds ratios (ORs) with 95% CIs.

### Statistical Analysis and Covariates

A series of logistic regression analyses was conducted to examine the associations between nicotine dependence and self-confidence in quitting with smoking cessation success. The outcome variable, “cessation success,” was binary (1=“quit” and 0=“not quit”), and ORs with 95% CIs were calculated to estimate the strength of the associations. These analyses were conducted both overall and separately according to tobacco product types (cigarette-only smokers, HTP-only users, and dual users) to explore potential differences in associations across tobacco use types.

First, participants in the high-dependence group were used as the reference to analyze the relationship between nicotine dependence and smoking cessation. The model evaluated whether those in the low-dependence group had higher ORs for achieving cessation. Subsequently, the relationship between self-confidence for quitting and smoking cessation rates was evaluated. Using participants in the low self-confidence group as the reference, the ORs for the high self-confidence group were estimated. Finally, the combined effects of nicotine dependence and self-confidence were analyzed, with the groups characterized by high and low self-confidence serving as the reference.

Three levels of adjustment were applied. Model 1 was adjusted for age (continuous) and sex (male or female). Model 2 was additionally adjusted for smoking duration (0‐2, 3‐4, 5‐9, 10‐19, 20‐29, or ≥30 y) and the number of previous smoking cessation attempts (0, 1, 2, 3, 4, 5, 6, 7, 8, 9, 10, or ≥11). Model 3 was further adjusted for nicotine dependence (low or high) and self-confidence for quitting (low or high) to account for potential mutual confounding factors. In all models, companies were incorporated as clusters. The conceptual framework is shown in Figure S1 in Multimedia Appendix 1.

All statistical analyses were performed using Stata version 19.0 (StataCorp). This study was reported in accordance with the American Psychological Association (APA) Journal Article Reporting Standards for Quantitative Research (JARS-Quant) [46].

### Additional Analyses for Missing Data

To assess the robustness of our findings, we conducted additional analyses. First, the Little test was used to assess whether the missing outcome data were missing completely at random, using all covariates included in the primary adjusted analyses (χ^2^_6_=41.77; *P*<.001). We also performed multiple imputations for missing outcome data using a logistic imputation model, with the outcome variable imputed and baseline covariates treated as regular variables. The imputation model included the same covariates as in the primary adjusted analyses, and 20 imputed datasets were generated. To improve model stability, the number of previous smoking cessation attempts was collapsed into 5 categories (0, 1, 2, 3, and ≥4) for the imputation procedure. Estimates were combined across imputations using the Rubin rules. Second, as a sensitivity analysis, all participants with missing outcome data were assumed to have failed smoking cessation, and the analyses were repeated using the same covariates as in the primary adjusted models.

### Ethical Considerations

This study was approved by the Research Ethics Committee of the Institutional Review Board of Kitasato University School of Allied Health Sciences (approval number: 2024-008-3). This retrospective cohort study was a secondary analysis of data from a company-implemented smoking cessation program in which the amount of nicotine patches or gum provided was adjusted according to the participants’ cigarette consumption. The program content, delivery, and operation were not modified for the purpose of this research, and no additional data were collected specifically for this study. In accordance with the ethics committee–approved protocol, the requirement for additional individual informed consent for this secondary analysis was waived, and an opt-out procedure was used. Information about the study was publicly disclosed on the institutional website, and participants were given the opportunity to decline participation or withdraw their data at any time. All data were anonymized or deidentified prior to analysis, and no personally identifiable information was included in the analytic dataset. Participants received no financial compensation or other incentives. We confirm that individual participants or users cannot be identified in any images included in the paper or Multimedia Appendix 1.

## Results

**Figure 3.**
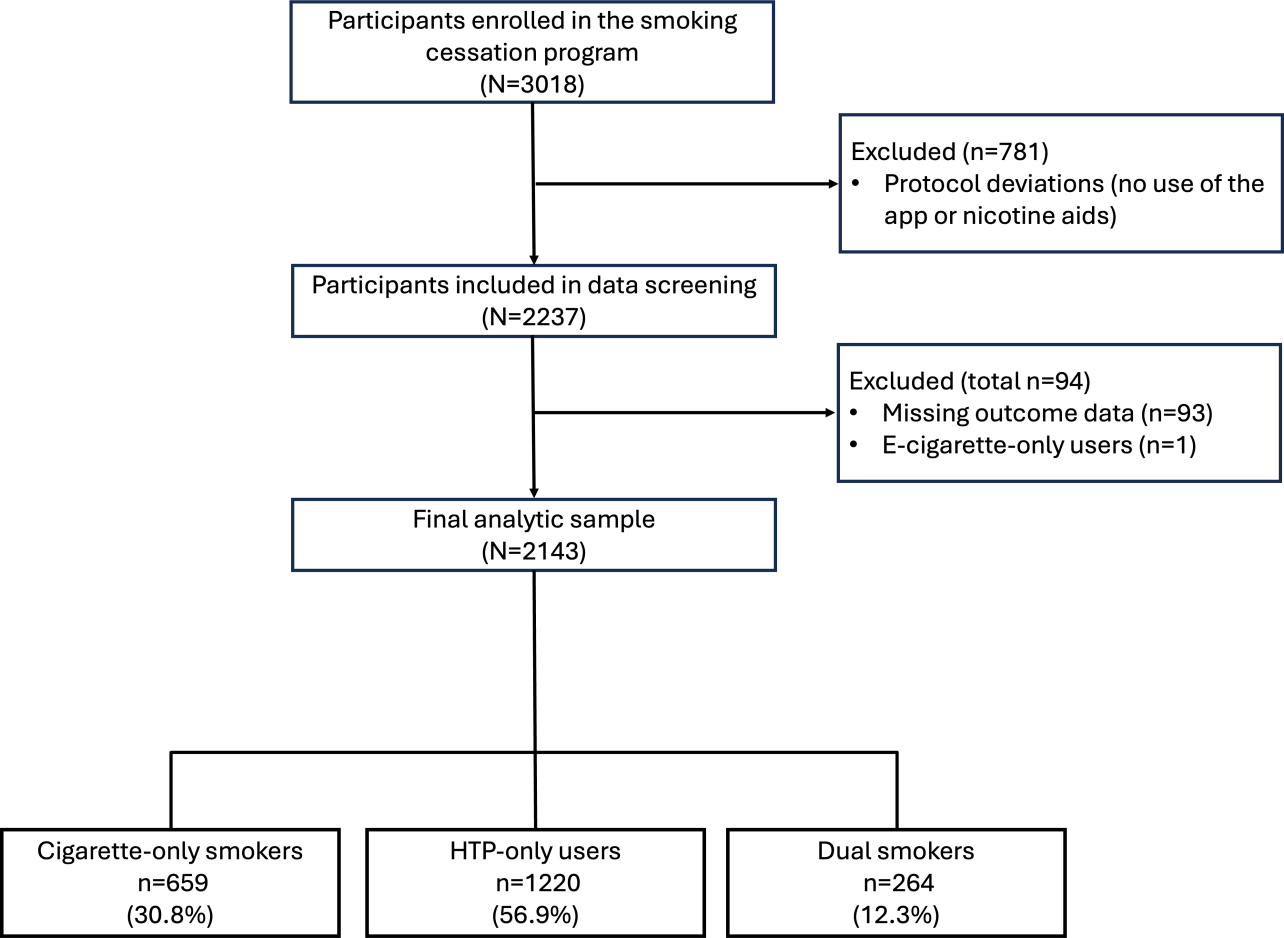
Flowchart showing participant eligibility, exclusions, data screening, and the final analytic sample in this retrospective cohort study using data from a company-implemented smoking cessation program conducted between May 2024 and March 2025. HTP: heated tobacco product.

The participant flowchart is presented in Figure 3. Overall, 3018 employed smokers were enrolled in the smoking cessation program, which combined the use of the digital peer-supported app and the use of nicotine gum or patches. After excluding 781 individuals who had never used the app, nicotine gum, or patches, and 93 individuals who had missing outcome data on smoking cessation at the end of the program, the final analytic sample included 2143 participants who adhered to the intervention and provided complete outcome data.

Participant characteristics, total and stratified by cessation status, are shown in Table 2. Compared with the cessation failure group, the cessation success group was older, had a higher proportion of HTP-only users, and demonstrated higher self-confidence for quitting, higher posting frequency in the digital peer-supported application, and more approvals from group members. In addition, the cessation success group tended to have a smoking history of 20 years or more, smoke fewer cigarettes per day, rate the importance of smoking cessation more highly, and show lower nicotine dependence. Characteristics stratified by nicotine dependence, self-confidence for quitting, and their combination are shown in Tables S1-S3 in Multimedia Appendix 1, respectively.

**Table 2.** Baseline characteristics of current smokers enrolled in a company-implemented smoking cessation program in Japan, overall and stratified by smoking cessation status—a retrospective cohort study of employees from 38 companies conducted from May 2024 to March 2025 (N=2143)^a^.

Characteristics	Total (N=2143)	Cessation success (n=1152)	Cessation failure (n=991)	*P* value
Age (y), mean (SD)	46.5 (10.9)	47.7 (10.4)	45.0 (11.3)	<.001
Sex, n (%)				.61
Male	1903 (88.8)	1027 (89.1)	876 (88.4)	
Female	209 (9.8)	111 (9.6)	98 (9.9)	
Others	31 (1.4)	14 (1.2)	17 (1.7)	
Type of cigarettes used, n (%)				<.001
Cigarette-only smokers	659 (30.8)	332 (28.8)	327 (33)	
Heated tobacco product–only users	1220 (56.9)	696 (60.4)	524 (52.9)	
Dual users	264 (12.3)	124 (10.8)	140 (14.1)	
Smoking duration (y), n (%)				.05
0‐2	51 (2.4)	24 (2.1)	27 (2.7)	
3‐4	68 (3.2)	31 (2.7)	37 (3.7)	
5‐9	190 (8.9)	97 (8.4)	93 (9.4)	
10‐19	391 (18.2)	191 (16.6)	200 (20.2)	
20‐29	673 (31.4)	369 (32)	304 (30.7)	
≥30	770 (35.9)	440 (38.2)	330 (33.3)	
Cigarettes/day, mean (SD)	17.8 (8.4)	17.4 (8.5)	18.1 (8.2)	.06
Number of prior smoking cessation attempts, n (%)				.85
0	666 (31.1)	361 (31.3)	305 (30.8)	
1	645 (30.1)	347 (30.1)	298 (30.1)	
2	355 (16.6)	197 (17.1)	158 (15.9)	
3	257 (12.0)	135 (11.7)	122 (12.3)	
≥4	220 (10.3)	112 (9.7)	108 (10.9)	
Provision patterns of nicotine gum or patches, n (%)				.32
A: 7 sheets of 20 cm² patches + 96 pieces of gum	1584 (73.9)	834 (72.4)	750 (75.7)	
B: 14 sheets of 10 cm² patches + 48 pieces of gum	491 (22.9)	284 (24.7)	207 (20.9)	
C: 14 sheets of 20 cm² patches + 14 sheets of 10 mg patches	32 (1.5)	15 (1.3)	17 (1.7)	
D: 20 sheets of 10 cm² patches	8 (0.4)	4 (0.3)	4 (0.4)	
E: 96 pieces of gum alone	28 (1.3)	15 (1.3)	13 (1.3)	
Importance of smoking cessation (score: 0‐10), mean (SD)	7 (2.4)	7.2 (2.3)	6.7 (2.4)	<.001
Nicotine dependence, n (%)				.07
High (≤30 min after waking)	1653 (77.1)	871 (75.6)	782 (78.9)	
Low (>30 min after waking)	490 (22.9)	281 (24.4)	209 (21.1)	
Self-confidence for quitting, n (%)				<.001
High (scores of 5‐10)	834 (38.9)	529 (45.9)	305 (30.8)	
Low (scores of 0‐4)	1309 (61.1)	623 (54.1)	686 (69.2)	
Posting frequency in the digital peer-supported app, mean (SD)	32.6 (78.4)	51.5 (98.7)	10.7 (33.1)	<.001
Posting approvals from group members, mean (SD)	54.8 (100.6)	88.1 (121.2)	16.0 (44.9)	<.001

a Data are presented as means and SDs for continuous variables and as numbers (percentages) for categorical variables. The Pearson chi-square test was used for categorical variables, and the Mann-Whitney *U* test was used for continuous variables, as appropriate. For presentation purposes, the number of previous smoking cessation attempts was collapsed into 5 categories (0, 1, 2, 3, and ≥4) in this table. In all regression analyses, the original detailed categories were used.

The association between nicotine dependence and smoking cessation success among all participants, according to tobacco product type, is presented in Table 3. Among all participants, smokers with low nicotine dependence showed a nonsignificantly higher likelihood of smoking cessation success compared to those with high dependence (281/490, 57.3% vs 871/1753, 52.7%, respectively). When stratified by tobacco product type, the association between lower nicotine dependence and cessation success remained significant among cigarette-only smokers (96/166, 57.8% vs 236/493, 47.9%, respectively). The adjusted OR was 1.59 (95% CI 1.07‐2.37) in model 3. However, no significant associations were observed among HTP-only and dual users.

**Table 3.** Odds ratios (ORs) for smoking cessation success according to nicotine dependence among current smokers enrolled in a company-implemented smoking cessation program in Japan—a retrospective cohort study of employees from 38 companies conducted from May 2024 to March 2025^a^.

Characteristics	Nicotine dependence	
	High (≤30 min after waking)	Low (>30 min after waking)
Overall		
Number of participants	1653	490
Smoking cessation success, n (%)	871 (52.7)	281 (57.3)
Model 1, OR (95% CI)^b^	1 (reference)	1.25 (1.05‐1.49)
Model 2, OR (95% CI)^c^	1 (reference)	1.22 (1.02‐1.46)
Model 3, OR (95% CI)^d^	1 (reference)	1.14 (0.96‐1.36)
Cigarette-only smokers		
Number of participants	493	166
Smoking cessation success, n (%)	236 (47.9)	96 (57.8)
Model 1, OR (95% CI)^b^	1 (reference)	1.66 (1.11‐2.47)
Model 2, OR (95% CI)^c^	1 (reference)	1.67 (1.11‐2.51)
Model 3, OR (95% CI)^d^	1 (reference)	1.59 (1.07‐2.37)
HTP^e^-only users		
Number of participants	952	268
Smoking cessation success, n (%)	541 (56.8)	155 (57.8)
Model 1, OR (95% CI)^b^	1 (reference)	1.07 (0.84‐1.36)
Model 2, OR (95% CI)^c^	1 (reference)	1.05 (0.81‐1.36)
Model 3, OR (95% CI)^d^	1 (reference)	0.97 (0.74‐1.26)
Dual users		
Number of participants	208	56
Smoking cessation success, n (%)	94 (45.2)	30 (53.6)
Model 1, OR (95% CI)^b^	1 (reference)	1.41 (0.74‐2.71)
Model 2, OR (95% CI)^c^	1 (reference)	1.38 (0.70‐2.70)
Model 3, OR (95% CI)^d^	1 (reference)	1.27 (0.68‐2.37)

a ORs and 95% CIs were estimated using logistic regression analyses. In all models, companies were incorporated as clusters.

b Adjusted for age and sex.

c Adjusted for age, sex, smoking duration, and the number of previous smoking cessation attempts.

d Adjusted for age, sex, smoking duration, the number of previous smoking cessation attempts, and self-confidence in quitting.

e HTP: heated tobacco product.

The association between self-confidence for quitting and cessation success among all participants and tobacco product types is presented in Table 4. Among all participants, the higher self-confidence group was associated with higher smoking cessation success rates: 47.6% (623/1309) in participants with low self-confidence and 63.4% (529/834) in those with high self-confidence. Compared with the low self-confidence group, the adjusted OR for cessation success in the high self-confidence group was 1.81 (95% CI 1.55‐2.12) in model 3. When stratified by tobacco product types, this positive association remained significant across all groups. Among cigarette-only smokers, the cessation success rate increased from 44.7% (177/396; low) to 58.9% (155/263; high), with an adjusted OR of 1.71 (95% CI 1.26‐2.31) for the high group in model 3. Similarly, among HTP-only users, the success rates were 51.4% (380/740; low) and 65.8% (316/480; high), and the adjusted OR was 1.78 (95% CI 1.44‐2.21) in model 3. A similar association was observed in dual users, with adjusted ORs of 2.95 (95% CI 1.58‐5.53) for the high group in model 3.

**Table 4.** Odds ratios (ORs) for smoking cessation success according to self-confidence in quitting among current smokers enrolled in a company-implemented smoking cessation program in Japan: a retrospective cohort study of employees from 38 companies conducted from May 2024 to March 2025^a^.

Characteristics	Self-confidence for quitting	
	Low (scores of 0-4)	High (scores of 5‐10)
Overall		
Number of participants	1309	834
Smoking cessation success, n (%)	623 (47.6)	529 (63.4)
Model 1, OR (95% CI)^b^	1 (reference)	1.84 (1.56‐2.18)
Model 2, OR (95% CI)^c^	1 (reference)	1.83 (1.57‐2.15)
Model 3, OR (95% CI)^d^	1 (reference)	1.81 (1.55‐2.12)
Cigarette-only smokers		
Number of participants	396	263
Smoking cessation success, n (%)	177 (44.7)	155 (58.9)
Model 1, OR (95% CI)^b^	1 (reference)	1.73 (1.28‐2.35)
Model 2, OR (95% CI)^c^	1 (reference)	1.76 (1.30‐2.40)
Model 3, OR (95% CI)^d^	1 (reference)	1.71 (1.26‐2.31)
HTP^e^-only users		
Number of participants	740	480
Smoking cessation success, n (%)	380 (51.4)	316 (65.8)
Model 1, OR (95% CI)^b^	1 (reference)	1.76 (1.41‐2.19)
Model 2, OR (95% CI)^c^	1 (reference)	1.78 (1.43‐2.21)
Model 3, OR (95% CI)^d^	1 (reference)	1.78 (1.44‐2.21)
Dual users		
Number of participants	173	91
Smoking cessation success, n (%)	66 (38.2)	58 (63.7)
Model 1, OR (95% CI)^b^	1 (reference)	2.58 (1.49‐4.49)
Model 2, OR (95% CI)^c^	1 (reference)	3.00 (1.60‐5.61)
Model 3, OR (95% CI)^d^	1 (reference)	2.95 (1.58‐5.53)

a ORs and 95% CIs were estimated using logistic regression analyses. In all models, companies were incorporated as clusters.

b Adjusted for age and sex.

c Adjusted for age, sex, smoking duration, and the number of previous smoking cessation attempts.

d Adjusted for age, sex, smoking duration, the number of previous smoking cessation attempts, and nicotine dependence.

e HTP: heated tobacco product.

**Figure 4.**
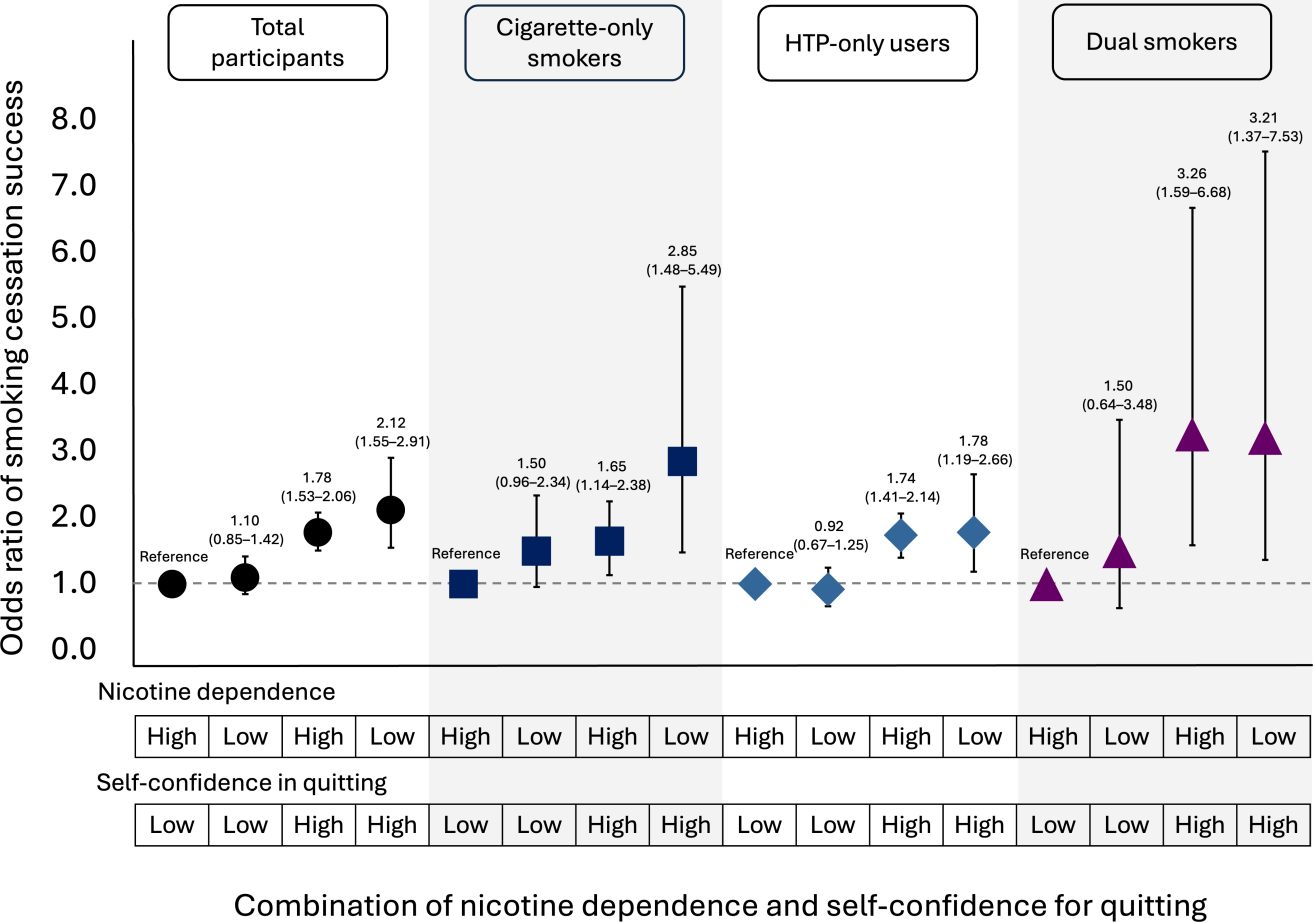
Adjusted odds ratios for smoking cessation success according to combined categories of nicotine dependence (ND) and self-confidence for quitting (SC) among current smokers enrolled in a company-implemented smoking cessation program in Japan—a retrospective cohort study of employees from 38 companies conducted from May 2024 to March 2025. Markers indicate adjusted odds ratios, and error bars indicate 95% CIs. Estimates are shown for all participants, cigarette-only smokers, heated tobacco product (HTP)-only users, and dual smokers. The reference group consisted of participants with high ND and low self-confidence for quitting.

The combined association of nicotine dependence and self-confidence for quitting with cessation success among all participants, according to tobacco product types, is shown in Table 5 and Figure 4. Among all participants, the smoking cessation success rate was highest in participants with low nicotine dependence and high self-confidence (159/241, 66%), followed by those with high dependence and high self-confidence (370/593, 62.4%), those with low dependence and low self-confidence (122/249, 49%), and those with high dependence and low self-confidence (501/1060, 47.3%). Using the high dependence and low self-confidence group as the reference, the adjusted ORs for cessation success were 1.78 (95% CI 1.53‐2.06) in the high dependence and high self-confidence group and 2.12 (95% CI 1.55‐2.91) in the low dependence and high self-confidence group in model 2. A similar pattern was observed when stratified by tobacco product types. Among cigarette-only smokers, those with low dependence and high self-confidence had the highest cessation success rate (52/79, 65.8%), with an adjusted OR of 2.85 (95% CI 1.48‐5.49), whereas those with high dependence and high self-confidence achieved a success rate of 56% (103/184), with an adjusted OR of 1.65 (95% CI 1.14‐2.38). Among HTP-only users, cessation success was also highest in the low dependence and high self-confidence group (91/137, 66.4%; OR 1.78, 95% CI 1.19‐2.66), followed by the high dependence and high self-confidence group (225/343, 65.6%; OR 1.74, 95% CI 1.41‐2.14). Among dual users, the same pattern was observed, with cessation rates of 64% (16/25) and 63.6% (42/66) and adjusted ORs of 3.21 (95% CI 1.37‐7.53) and 3.26 (95% CI 1.59‐6.68) for the low dependence and high self-confidence group and high dependence and high self-confidence group, respectively.

Among the 2237 participants eligible for outcome assessment after excluding those who never used the intervention components, 93 (4.2%) had missing outcome data. Little’s test was significant, indicating that the missing outcome data were not missing completely at random. We, therefore, conducted additional analyses using multiple imputation (Tables S4-S6 in Multimedia Appendix 1) and a sensitivity analysis in which all participants with missing outcome data were assumed to have failed smoking cessation (Tables S7-S9 in Multimedia Appendix 1). In both additional analyses, the overall pattern of results was consistent with that of the primary complete-case analysis, with no material changes in the point estimates or 95% CIs (Tables S4-S9 in Multimedia Appendix 1). Baseline characteristics under the assumption that participants with missing outcome data had failed smoking cessation and those according to inclusion in the analytic sample or exclusion due to missing outcome data are summarized in Tables S10 and S11 in Multimedia Appendix 1, respectively.

**Table 5.** Odds ratios (ORs) for smoking cessation success according to combined categories of nicotine dependence and self-confidence in quitting among current smokers enrolled in a company-implemented smoking cessation program in Japan—a retrospective cohort study of employees from 38 companies conducted from May 2024 to March 2025^a^.

Characteristics	Combination of nicotine dependence and self-confidence for quitting			
	High dependence and low self-confidence	Low dependence and low self-confidence	High dependence and high self-confidence	Low dependence and high self-confidence
Overall				
Number of participants	1060	249	593	241
Smoking cessation success, n (%)	501 (47.3)	122 (49)	370 (62.4)	159 (66)
Model 1, OR (95% CI)^b^	1 (reference)	1.10 (0.86‐1.40)	1.77 (1.51‐2.08)	2.17 (1.57‐3.01)
Model 2, OR (95% CI)^c^	1 (reference)	1.10 (0.85‐1.42)	1.78 (1.53‐2.06)	2.12 (1.55‐2.91)
Cigarette-only smokers				
Number of participants	309	87	184	79
Smoking cessation success, n (%)	133 (43)	44 (50.6)	103 (56)	52 (65.8)
Model 1, OR (95% CI)^b^	1 (reference)	1.46 (0.94‐2.29)	1.60 (1.14‐2.25)	2.78 (1.43‐5.39)
Model 2, OR (95% CI)^c^	1 (reference)	1.50 (0.96‐2.34)	1.65 (1.14‐2.38)	2.85 (1.48‐5.49)
HTP^d^-only users				
Number of participants	609	131	343	137
Smoking cessation success, n (%)	316 (51.9)	64 (48.9)	225 (65.6)	91 (66.4)
Model 1, OR (95% CI)^b^	1 (reference)	0.92 (0.67‐1.25)	1.70 (1.40‐2.07)	1.81 (1.22‐2.69)
Model 2, OR (95% CI)^c^	1 (reference)	0.92 (0.67‐1.25)	1.74 (1.41‐2.14)	1.78 (1.19‐2.66)
Dual users				
Number of participants	142	31	66	25
Smoking cessation success, n (%)	52 (36.6)	14 (45.2)	42 (63.6)	16 (64)
Model 1, OR (95% CI)^b^	1 (reference)	1.47 (0.66‐3.25)	2.75 (1.47‐5.13)	2.83 (1.27‐6.35)
Model 2, OR (95% CI)^c^	1 (reference)	1.50 (0.64‐3.48)	3.26 (1.59‐6.68)	3.21 (1.37‐7.53)

a ORs and 95% CIs were estimated using logistic regression analyses. In all models, companies were incorporated as clusters.

b Adjusted for age and sex.

c Adjusted for age, sex, smoking duration, and the number of previous smoking cessation attempts.

d HTP: heated tobacco product.

## Discussion

### Principal Results

This study examined the independent and combined associations of nicotine dependence and self-confidence in quitting with smoking cessation success across cigarette-only smokers, HTP-only users, and dual users in a company-implemented smoking cessation program using a digital peer-supported app and nicotine aids. Overall, higher self-confidence in quitting was consistently associated with greater smoking cessation success across tobacco product types, whereas lower nicotine dependence was associated with greater cessation success only among cigarette-only smokers. In addition, when nicotine dependence and self-confidence were considered jointly, groups with high self-confidence showed the most favorable cessation outcomes regardless of nicotine dependence level.

### Interpretation and Comparison With Previous Studies

Our findings align with previous studies on cigarette-only smokers, which have consistently reported an association between nicotine dependence and smoking cessation [11,16-20]. However, no statistically significant association was observed among HTP-only and dual users in the present study. Although some app-based intervention studies have examined cessation outcomes according to tobacco product type, the association between nicotine dependence and smoking cessation success by tobacco product type has received limited attention [10,24,25]. In this context, our study extends previous research conducted among cigarette-only smokers [11,16-20] by stratifying participants according to tobacco product type and estimating the association between nicotine dependence and smoking cessation success in an app-based intervention.

Furthermore, our findings that higher self-confidence for quitting contributed to successful quitting are consistent with a meta-analysis [21], which highlights self-confidence for quitting as a key psychological determinant of smoking cessation. In addition, in the context of a digital mobile health app, a previous study examined psychological empowerment [47], whereas our study focused on self-confidence for quitting, which is a related but conceptually distinct construct.

One possible explanation for the nonsignificant association between nicotine dependence and cessation success among HTP-only and dual users may be differences in underlying motivations and perceptions compared with cigarette-only smokers [48,49]. For example, a study in the United States using cross-sectional data from 2470 young adults identified “fashion” as a notable perceived attribute of HTPs [48]. Another qualitative study involving adults in Switzerland and Japan found that many perceived the packaging of HTPs as appealing and described HTPs as sophisticated, high-tech, or aspirational [49].

Furthermore, the digital peer-supported app may have contributed to self-confidence in quitting through its team-based, interactive features. Consistent with this interpretation, a pilot study using the same digital peer-supported app found that participants regarded the team system and challenge-tracking features as particularly useful and reported that the app provided emotional support and motivation through social comparison [50].

The tobacco product–specific findings in this study, particularly among dual users, should be interpreted cautiously because the corresponding estimates were less precise than those in the overall sample. Nevertheless, the overall findings suggest that both nicotine dependence and self-confidence for quitting, alongside tobacco product type, may be useful considerations in tailoring smoking cessation support in similar workplace app-based settings.

### Limitations

This study has some limitations. First, the lack of a control group limited the ability to attribute the observed smoking cessation success solely to the app. However, in our proof-of-concept trial, the group using both smoking cessation aids and the digital peer-supported app achieved a higher smoking cessation success rate than the group using only smoking cessation aids [9]. Second, the outcomes were self-reported and not biochemically verified, which may have affected the accuracy of smoking cessation status. This may have led to an overestimation of the absolute quit rates, as some participants may have reported successful cessation due to social desirability or a desire to present themselves favorably. In addition, participants with higher self-confidence for quitting may have been more likely to report success, which could have particularly affected the magnitude of the observed quit rates among those with high self-confidence. However, although the lack of biochemical verification may have influenced the absolute success rates, it is less likely to have substantially altered the relative differences across tobacco product groups or nicotine dependence levels. Although expert consensus indicates that biochemical verification is often impractical and unnecessary in similar studies [51], future research should consider incorporating such methods to enhance the validity of the findings. Third, the sample size was determined by the number of eligible participants available during the study period rather than by an a priori sample size calculation. Although precision was assessed using 95% CIs, the study may have had limited statistical power, especially for subgroup and additional analyses. Fourth, we did not account for potential confounding variables, including sociodemographic characteristics (eg, occupation, education, and income), smartphone usage patterns, adverse events, continued nicotine product use, or a history of smoking cessation [13]. Additionally, other potentially relevant factors such as alcohol or substance use, mental health conditions (eg, depression or anxiety), chronic diseases (eg, asthma or chronic obstructive pulmonary disease), and behavioral stages of change (eg, precontemplation, contemplation, preparation, and action) were not measured. Furthermore, nicotine dependence and self-confidence for quitting were assessed only at baseline and may have changed during follow-up; therefore, the analyses could not account for time-varying changes in these factors.

Fifth, activity-related indicators within the digital peer-supported app were limited to basic posting frequency and duration. Future evaluations should incorporate detailed in-app behavioral metrics (eg, message content, peer engagement quality, and feedback responsiveness) to better assess the mechanisms of behavior change. Finally, this study sample consisted primarily of male employees from large Japanese companies, which limits the generalizability of our findings to other populations, including women and nonworking individuals. Moreover, because the participants were employees enrolled in a company-based smoking cessation program, they may have had greater organizational support for cessation than the general population. They may also have been more motivated to quit than smokers not enrolled in such programs, which may have introduced selection bias. These factors may also have contributed to the different distribution of cigarette-only smokers and HTP-only users observed in our sample compared with national estimates, thereby limiting generalizability.

### Conclusions

By distinguishing cigarette-only smokers, HTP-only users, and dual users in a workplace cessation program, this study provides novel, product-specific evidence on how nicotine dependence and self-confidence relate to cessation success. By examining tobacco user groups, this study extends prior research beyond cigarette-only smokers and suggests that self-confidence may predict cessation across tobacco product types, whereas the role of nicotine dependence may be product-specific. These findings may inform tailored, scalable smoking-cessation support in workplaces; however, they should be interpreted with caution because cessation outcomes were self-reported and not biochemically verified. Further research, including randomized controlled trials with objective outcome verification, is needed to confirm these findings.

## Supplementary material

10.2196/84792 Multimedia Appendix 1 Baseline characteristics and additional analyses.

## Abbreviations

APA: American Psychological Association
HTP: heated tobacco product
JARS-Quant: Journal Article Reporting Standards for Quantitative Research
OR: odds ratio
