# Supplementary material for: Nicotine Dependence and Quit Self-Confidence in a Smoking Cessation Program Using a Group-Based Digital Peer-Supported App and Cigarette Consumption–Adjusted Nicotine Aids Among Japanese Workers: Retrospective Cohort Study
Source: J Med Internet Res. 2026 May 19;28:e84792. doi: 10.2196/84792 (PMC13186532; doi:10.2196/84792)
Supplement: Multimedia Appendix 1 [file jmir-v28-e84792-s001.docx]

**Supplementary Table 1. Baseline characteristics of current smokers enrolled in a company-implemented smoking cessation program in Japan, overall and stratified by nicotine dependence: a retrospective observational study of employees from 38 companies conducted from May 2024 to March 2025.**

|  |  |  | | | | Nicotine dependence | | | | | | | |  |
| --- | --- | --- | --- | --- | --- | --- | --- | --- | --- | --- | --- | --- | --- | --- |
|  |  | Total | | | | High  (≤30 min after waking) | | | | Low  (>30 min after waking) | | | |  |
|  |  | (n=2,143) | | | | (n=1,653) | | | | (n=490) | | | | *P*-value |
| Age, years | | 46.5 | ± | 10.9 |  | 46.7 | ± | 10.9 |  | 45.6 | ± | 10.7 |  | .042 |
| Sex, *n* (%) | |  |  |  |  |  |  |  |  |  |  |  |  | .16 |
|  | Male | 1903 | ( | 88.8 | ) | 1,466 | ( | 88.7 | ) | 437 | ( | 89.2 | ) |  |
|  | Female | 209 | ( | 9.8 | ) | 167 | ( | 10.1 | ) | 42 | ( | 8.6 | ) |  |
|  | Others | 31 | ( | 1.4 | ) | 20 | ( | 1.2 | ) | 11 | ( | 2.2 | ) |  |
| Type of Cigarettes Used, *n* (%) | |  |  |  |  |  |  |  |  |  |  |  |  | .22 |
|  | Cigarette-only smokers | 659 | ( | 30.8 | ) | 493 | ( | 29.8 | ) | 166 | ( | 33.9 | ) |  |
|  | HTP-only users | 1220 | ( | 56.9 | ) | 952 | ( | 57.6 | ) | 268 | ( | 54.7 | ) |  |
|  | Dual users | 264 | ( | 12.3 | ) | 208 | ( | 12.6 | ) | 56 | ( | 11.4 | ) |  |
| Smoking duration (years), *n* (%) | |  |  |  |  |  |  |  |  |  |  |  |  | .008 |
|  | 0–2 | 51 | ( | 2.4 | ) | 35 | ( | 2.1 | ) | 16 | ( | 3.3 | ) |  |
|  | 3–4 | 68 | ( | 3.2 | ) | 51 | ( | 3.1 | ) | 17 | ( | 3.5 | ) |  |
|  | 5–9 | 190 | ( | 8.9 | ) | 140 | ( | 8.5 | ) | 50 | ( | 10.2 | ) |  |
|  | 10–29 | 391 | ( | 18.2 | ) | 285 | ( | 17.2 | ) | 106 | ( | 21.6 | ) |  |
|  | 20–29 | 673 | ( | 31.4 | ) | 515 | ( | 31.2 | ) | 158 | ( | 32.2 | ) |  |
|  | 30+ | 770 | ( | 35.9 | ) | 627 | ( | 37.9 | ) | 143 | ( | 29.2 | ) |  |
| Cigarettes/day | | 17.8 | ± | 8.4 |  | 19.2 | ± | 8.5 |  | 12.9 | ± | 5.8 |  | <.001 |
| Number of prior smoking cessation attempts, *n* (%) | |  |  |  |  |  |  |  |  |  |  |  |  | .11 |
|  | 0 | 666 | ( | 31.1 | ) | 514 | ( | 31.1 | ) | 152 | ( | 31 | ) |  |
|  | 1 | 645 | ( | 30.1 | ) | 518 | ( | 31.3 | ) | 127 | ( | 25.9 | ) |  |
|  | 2 | 355 | ( | 16.6 | ) | 260 | ( | 15.7 | ) | 95 | ( | 19.4 | ) |  |
|  | 3 | 257 | ( | 12 | ) | 192 | ( | 11.6 | ) | 65 | ( | 13.3 | ) |  |
|  | 4+ | 220 | ( | 10.3 | ) | 169 | ( | 10.2 | ) | 51 | ( | 10.4 | ) |  |
| Provision patterns of nicotine gums or patch, *n* (%) | |  |  |  |  |  |  |  |  |  |  |  |  | <.001 |
|  | A: 7 sheets of 20-cm² patches + 96 pieces of gum | 1584 | ( | 73.9 | ) | 1,357 | ( | 82.1 | ) | 227 | ( | 46.3 | ) |  |
|  | B: 14 sheets of 10-cm² patches + 48 pieces of gum | 491 | ( | 22.9 | ) | 252 | ( | 15.2 | ) | 239 | ( | 48.8 | ) |  |
|  | C: 14 sheets of 20-cm² patches + 14 sheets of 10-mg patches | 32 | ( | 1.5 | ) | 27 | ( | 1.6 | ) | 5 | ( | 1 | ) |  |
|  | D: 20 sheets of 10-cm² patches | 8 | ( | 0.4 | ) | 7 | ( | 0.4 | ) | 1 | ( | 0.2 | ) |  |
|  | E: 96 pieces of gum alone | 28 | ( | 1.3 | ) | 10 | ( | 0.6 | ) | 18 | ( | 3.7 | ) |  |
| Importance of smoking cessation (score: 0–10) | | 7 | ± | 2.4 |  | 7 | ± | 2.4 |  | 6.9 | ± | 2.4 |  | .53 |
| High self-confidence for quitting | |  |  |  |  |  |  |  |  |  |  |  |  | <.001 |
|  | High (scores of 5−10) | 834 | ( | 38.9 | ) | 593 | ( | 35.9 | ) | 241 | ( | 49.2 | ) |  |
|  | Low (scores of 0−4) | 1309 | ( | 61.1 | ) | 1,060 | ( | 64.1 | ) | 249 | ( | 50.8 | ) |  |
| Posting frequency in the digital peer-supported app | | 32.6 | ± | 78.4 |  | 32.4 | ± | 84.7 |  | 33.5 | ± | 52.3 |  | .79 |
| Posting approvals from group members | | 54.8 | ± | 100.6 |  | 54.1 | ± | 103.8 |  | 57.3 | ± | 88.9 |  | .53 |

*Note:* Data are presented as means and standard deviations (SDs) for continuous variables and as numbers (percentages) for categorical variables. The Pearson chi-square test was used for categorical variables, as appropriate. The Mann–Whitney U test was used for continuous variables. HTP: heated tobacco product. For presentation purposes, the number of previous smoking cessation attempts was collapsed into 5 categories (0, 1, 2, 3, and ≥4).

**Supplementary Table 2. Baseline characteristics of current smokers enrolled in a company-implemented smoking cessation program in Japan, overall and stratified by self-confidence for quitting: a retrospective observational study of employees from 38 companies conducted from May 2024 to March 2025.**

|  |  |  | | | | Self-confidence for quitting | | | | | | | |  |
| --- | --- | --- | --- | --- | --- | --- | --- | --- | --- | --- | --- | --- | --- | --- |
|  |  | Total | | | | Low  (scores of 0−4) | | | | High  (scores of 5−10) | | | |  |
|  |  | (n=2143) | | | | (n=1309) | | | | (n=834) | | | | *P*-value |
| Age, years | | 46.5 | ± | 10.9 |  | 45.7 | ± | 10.8 |  | 47.7 | ± | 10.9 |  | <.001 |
| Sex, *n* (%) | |  |  |  |  |  |  |  |  |  |  |  |  | .096 |
|  | Male | 1903 | ( | 88.8 | ) | 1,147 | ( | 87.6 | ) | 756 | ( | 90.6 | ) |  |
|  | Female | 209 | ( | 9.8 | ) | 141 | ( | 10.8 | ) | 68 | ( | 8.2 | ) |  |
|  | Others | 31 | ( | 1.4 | ) | 21 | ( | 1.6 | ) | 10 | ( | 1.2 | ) |  |
| Type of Cigarettes Used, *n* (%) | |  |  |  |  |  |  |  |  |  |  |  |  | .28 |
|  | Cigarette-only smokers | 659 | ( | 30.8 | ) | 396 | ( | 30.3 | ) | 263 | ( | 31.5 | ) |  |
|  | HTP-only users | 1220 | ( | 56.9 | ) | 740 | ( | 56.5 | ) | 480 | ( | 57.6 | ) |  |
|  | Dual users | 264 | ( | 12.3 | ) | 173 | ( | 13.2 | ) | 91 | ( | 10.9 | ) |  |
| Smoking duration (years), *n* (%) | |  |  |  |  |  |  |  |  |  |  |  |  | <.001 |
|  | 0–2 | 51 | ( | 2.4 | ) | 18 | ( | 1.4 | ) | 33 | ( | 4 | ) |  |
|  | 3–4 | 68 | ( | 3.2 | ) | 43 | ( | 3.3 | ) | 25 | ( | 3 | ) |  |
|  | 5–9 | 190 | ( | 8.9 | ) | 114 | ( | 8.7 | ) | 76 | ( | 9.1 | ) |  |
|  | 10–29 | 391 | ( | 18.2 | ) | 249 | ( | 19 | ) | 142 | ( | 17 | ) |  |
|  | 20–29 | 673 | ( | 31.4 | ) | 436 | ( | 33.3 | ) | 237 | ( | 28.4 | ) |  |
|  | 30+ | 770 | ( | 35.9 | ) | 449 | ( | 34.3 | ) | 321 | ( | 38.5 | ) |  |
| Cigarettes/day | | 17.8 | ± | 8.4 |  | 18.6 | ± | 8.3 |  | 16.5 | ± | 8.4 |  | <.001 |
| Number of prior smoking cessation attempts, *n* (%) | |  |  |  |  |  |  |  |  |  |  |  |  | <.001 |
|  | 0 | 666 | ( | 31.1 | ) | 445 | ( | 34.0 | ) | 221 | ( | 26.5 | ) |  |
|  | 1 | 645 | ( | 30.1 | ) | 375 | ( | 28.6 | ) | 270 | ( | 32.4 | ) |  |
|  | 2 | 355 | ( | 16.6 | ) | 194 | ( | 14.8 | ) | 161 | ( | 19.3 | ) |  |
|  | 3 | 257 | ( | 12 | ) | 154 | ( | 11.8 | ) | 103 | ( | 12.4 | ) |  |
|  | 4+ | 220 | ( | 10.3 | ) | 141 | ( | 10.8 | ) | 79 | ( | 9.5 | ) |  |
| Provision patterns of nicotine gums or patch, *n* (%) | |  |  |  |  |  |  |  |  |  |  |  |  | <.001 |
|  | A: 7 sheets of 20-cm² patches + 96 pieces of gum | 1584 | ( | 73.9 | ) | 1,017 | ( | 77.7 | ) | 567 | ( | 68 | ) |  |
|  | B: 14 sheets of 10-cm² patches + 48 pieces of gum | 491 | ( | 22.9 | ) | 257 | ( | 19.6 | ) | 234 | ( | 28.1 | ) |  |
|  | C: 14 sheets of 20-cm² patches + 14 sheets of 10-mg patches | 32 | ( | 1.5 | ) | 21 | ( | 1.6 | ) | 11 | ( | 1.3 | ) |  |
|  | D: 20 sheets of 10-cm² patches | 8 | ( | 0.4 | ) | 1 | ( | 0.1 | ) | 7 | ( | 0.8 | ) |  |
|  | E: 96 pieces of gum alone | 28 | ( | 1.3 | ) | 13 | ( | 1 | ) | 15 | ( | 1.8 | ) |  |
| Importance of smoking cessation (score: 0–10) | | 7 | ± | 2.4 |  | 6.7 | ± | 2.4 |  | 7.4 | ± | 2.2 |  | <.001 |
| Nicotine dependence | |  |  |  |  |  |  |  |  |  |  |  |  | <.001 |
|  | High (≤30 min after waking) | 1653 | ( | 77.1 | ) | 1,060 | ( | 81 | ) | 593 | ( | 71.1 | ) |  |
|  | Low (>30 min after waking) | 490 | ( | 22.9 | ) | 249 | ( | 19 | ) | 241 | ( | 28.9 | ) |  |
| Posting frequency in the digital peer-supported app | | 32.6 | ± | 78.4 |  | 32.2 | ± | 91.3 |  | 33.3 | ± | 52.3 |  | .76 |
| Posting approvals from group members | | 54.8 | ± | 100.6 |  | 53.2 | ± | 103 |  | 57.2 | ± | 96.7 |  | .37 |

*Note:* Data are presented as means and standard deviations (SDs) for continuous variables and as numbers (percentages) for categorical variables. The Pearson chi-square test was used for categorical variables, as appropriate. The Mann–Whitney U test was used for continuous variables. HTP: heated tobacco product. For presentation purposes, the number of previous smoking cessation attempts was collapsed into 5 categories (0, 1, 2, 3, and ≥4).

**Supplementary Table 3. Baseline characteristics of current smokers enrolled in a company-implemented smoking cessation program in Japan, overall and stratified by the combination of nicotine dependence and self-confidence for quitting: a retrospective observational study of employees from 38 companies conducted from May 2024 to March 2025.**

|  |  | Combination of nicotine dependence and self-confidence for quitting | | | | | | | | | | | | | | | |  |  |
| --- | --- | --- | --- | --- | --- | --- | --- | --- | --- | --- | --- | --- | --- | --- | --- | --- | --- | --- | --- |
|  |  | High dependence and low self-confidence | | | | High dependence and high self-confidence | | | | Low dependence and low self-confidence | | | | Low dependence and high self-confidence | | | |  |  |
|  |  | (n=1,060) | | | | (n=593) | | | | (n=249) | | | | (n=241) | | | |  | *P*-value |
| Age, years | | 45.9 | ± | 10.9 |  | 48.3 | ± | 10.9 |  | 45.1 | ± | 10.6 |  | 46.1 | ± | 10.8 |  |  | <.001 |
| Sex, *n* (%) | |  |  |  |  |  |  |  |  |  |  |  |  |  |  |  |  |  | .093 |
|  | Male | 931 | ( | 87.8 | ) | 535 | ( | 90.2 | ) | 216 | ( | 86.7 | ) | 221 | ( | 91.7 | ) |  |  |
|  | Female | 116 | ( | 10.9 | ) | 51 | ( | 8.6 | ) | 25 | ( | 10 | ) | 17 | ( | 7.1 | ) |  |  |
|  | Others | 13 | ( | 1.2 | ) | 7 | ( | 1.2 | ) | 8 | ( | 3.2 | ) | 3 | ( | 1.2 | ) |  |  |
| Type of Cigarettes Used, *n* (%) | |  |  |  |  |  |  |  |  |  |  |  |  |  |  |  |  |  | .42 |
|  | Cigarette-only smokers | 309 | ( | 29.2 | ) | 184 | ( | 31.0 | ) | 87 | ( | 34.9 | ) | 79 | ( | 32.8 | ) |  |  |
|  | HTP-only users | 609 | ( | 57.5 | ) | 343 | ( | 57.8 | ) | 131 | ( | 52.6 | ) | 137 | ( | 56.8 | ) |  |  |
|  | Dual users | 142 | ( | 13.4 | ) | 66 | ( | 11.1 | ) | 31 | ( | 12.4 | ) | 25 | ( | 10.4 | ) |  |  |
| Smoking duration (years), *n* (%) | |  |  |  |  |  |  |  |  |  |  |  |  |  |  |  |  |  | <.001 |
|  | 0–2 | 12 | ( | 1.1 | ) | 23 | ( | 3.9 | ) | 6 | ( | 2.4 | ) | 10 | ( | 4.1 | ) |  |  |
|  | 3–4 | 35 | ( | 3.3 | ) | 16 | ( | 2.7 | ) | 8 | ( | 3.2 | ) | 9 | ( | 3.7 | ) |  |  |
|  | 5–9 | 92 | ( | 8.7 | ) | 48 | ( | 8.1 | ) | 22 | ( | 8.8 | ) | 28 | ( | 11.6 | ) |  |  |
|  | 10–29 | 195 | ( | 18.4 | ) | 90 | ( | 15.2 | ) | 54 | ( | 21.7 | ) | 52 | ( | 21.6 | ) |  |  |
|  | 20–29 | 352 | ( | 33.2 | ) | 163 | ( | 27.5 | ) | 84 | ( | 33.7 | ) | 74 | ( | 30.7 | ) |  |  |
|  | 30+ | 374 | ( | 35.3 | ) | 253 | ( | 42.7 | ) | 75 | ( | 30.1 | ) | 68 | ( | 28.2 | ) |  |  |
| Cigarettes/day | | 19.8 | ± | 8.3 |  | 18.2 | ± | 8.7 |  | 13.4 | ± | 5.8 |  | 12.4 | ± | 5.8 |  |  | <.001 |
| Number of prior smoking cessation attempts, *n* (%) | |  |  |  |  |  |  |  |  |  |  |  |  |  |  |  |  |  |  |
|  | 0 | 359 | ( | 33.9 | ) | 155 | ( | 26.1 | ) | 86 | ( | 34.5 | ) | 66 | ( | 27.4 | ) |  | .009 |
|  | 1 | 314 | ( | 29.6 | ) | 204 | ( | 34.4 | ) | 61 | ( | 24.5 | ) | 66 | ( | 27.4 | ) |  |  |
|  | 2 | 151 | ( | 14.2 | ) | 109 | ( | 18.4 | ) | 43 | ( | 17.3 | ) | 52 | ( | 21.6 | ) |  |  |
|  | 3 | 123 | ( | 11.6 | ) | 69 | ( | 11.6 | ) | 31 | ( | 12.4 | ) | 34 | ( | 14.1 | ) |  |  |
|  | 4+ | 113 | ( | 10.7 | ) | 56 | ( | 9.4 | ) | 28 | ( | 11.2 | ) | 23 | ( | 9.5 | ) |  |  |
| Provision patterns of nicotine gums or patch, *n* (%) | |  |  |  |  |  |  |  |  |  |  |  |  |  |  |  |  |  | <.001 |
|  | A: 7 sheets of 20-cm² patches + 96 pieces of gum | 893 | ( | 84.2 | ) | 464 | ( | 78.2 | ) | 124 | ( | 49.8 | ) | 103 | ( | 42.7 | ) |  |  |
|  | B: 14 sheets of 10-cm² patches + 48 pieces of gum | 141 | ( | 13.3 | ) | 111 | ( | 18.7 | ) | 116 | ( | 46.6 | ) | 123 | ( | 51 | ) |  |  |
|  | C: 14 sheets of 20-cm² patches + 14 sheets of 10-mg patches | 18 | ( | 1.7 | ) | 9 | ( | 1.5 | ) | 3 | ( | 1.2 | ) | 2 | ( | 0.8 | ) |  |  |
|  | D: 20 sheets of 10-cm² patches | 1 | ( | 0.1 | ) | 6 | ( | 1 | ) | 0 | ( | 0 | ) | 1 | ( | 0.4 | ) |  |  |
|  | E: 96 pieces of gum alone | 7 | ( | 0.7 | ) | 3 | ( | 0.5 | ) | 6 | ( | 2.4 | ) | 12 | ( | 5 | ) |  |  |
| Importance of smoking cessation (score: 0–10) | | 6.7 | ± | 2.5 |  | 7.5 | ± | 2.1 |  | 6.6 | ± | 2.4 |  | 7.2 | ± | 2.4 |  |  | <.001 |
| Posting frequency in the digital peer-supported app | | 33.1 | ± | 99.8 |  | 31.2 | ± | 46.6 |  | 28.6 | ± | 36.9 |  | 38.6 | ± | 64.1 |  |  | .51 |
| Posting approvals from group members | | 54.1 | ± | 109.1 |  | 53.9 | ± | 93.9 |  | 49.4 | ± | 71.7 |  | 65.4 | ± | 103.2 |  |  | .32 |

*Note:* Data are presented as means and standard deviations (SDs) for continuous variables and as numbers (percentages) for categorical variables. Continuous variables were compared using the Kruskal–Wallis test for the four groups. Categorical and binary variables were compared using Pearson’s chi-square test. HTP: heated tobacco product. For presentation purposes, the number of previous smoking cessation attempts was collapsed into 5 categories (0, 1, 2, 3, and ≥4).

**Supplementary Table 4. Multiple imputation analysis of odds ratios for smoking cessation success according to nicotine dependence among current smokers enrolled in a company-implemented smoking cessation program in Japan: a retrospective cohort study of employees from 38 companies conducted from May 2024 to March 2025.**

|  | Nicotine dependence | |
| --- | --- | --- |
|  | High  (≤30 min after waking) | Low  (>30 min after waking) |
| **Total participants** |  |  |
| Model 1 ^a^ | 1 (reference) | **1.25 (1.04–1.49)** |
| Model 2 ^b^ | 1 (reference) | **1.22 (1.01–1.46)** |
| Model 3 ^c^ | 1 (reference) | 1.13 (0.95–1.35) |
| **Cigarette-only smokers** |  |  |
| Model 1 ^a^ | 1 (reference) | **1.62 (1.09–2.42)** |
| Model 2 ^b^ | 1 (reference) | **1.58 (1.04–2.39)** |
| Model 3 ^c^ | 1 (reference) | 1.50 (0.99–2.26) |
| **HTP-only users** |  |  |
| Model 1 ^a^ | 1 (reference) | 1.08 (0.85–1.36) |
| Model 2 ^b^ | 1 (reference) | 1.05 (0.81–1.35) |
| Model 3 ^c^ | 1 (reference) | 0.96 (0.73–1.26) |
| **Dual users** |  |  |
| Model 1 ^a^ | 1 (reference) | 1.40 (0.73–2.67) |
| Model 2 ^b^ | 1 (reference) | 1.39 (0.71–2.74) |
| Model 3 ^c^ | 1 (reference) | 1.26 (0.68–2.36) |

Note: Odds ratios (ORs) and 95% confidence intervals (CIs) were estimated using logistic regression analyses. Missing outcome data were handled using multiple imputation, and 20 imputed datasets were generated. For multiple imputation, the number of previous smoking cessation attempts was modeled using collapsed categories (0, 1, 2, 3, and ≥4) to improve model stability.

Abbreviations: OR, odds ratio; CI, confidence interval; HTP, heated tobacco product.

^a^ Adjusted for age and sex.

^b^ Adjusted for age, sex, smoking duration, and number of previous smoking cessation attempts.

^c^ Adjusted for age, sex, smoking duration, number of previous smoking cessation attempts, and self-confidence for quitting.

In all models, companies were incorporated as clusters.

**Supplementary Table 5. Multiple imputation analysis of odds ratios for smoking cessation success according to self-confidence for quitting among current smokers enrolled in a company-implemented smoking cessation program in Japan: a retrospective cohort study of employees from 38 companies conducted from May 2024 to March 2025.**

|  | Self-confidence for quitting | |
| --- | --- | --- |
|  | Low  (scores of 0−4) | High  (scores of 5−10) |
| **Total participants** |  |  |
| Model 1 ^a^ | 1 (reference) | **1.85 (1.55–2.19)** |
| Model 2 ^b^ | 1 (reference) | **1.82 (1.54–2.15)** |
| Model 3 ^c^ | 1 (reference) | **1.80 (1.53–2.12)** |
| **Cigarette-only smokers** |  |  |
| Model 1 ^a^ | 1 (reference) | **1.73 (1.28–2.35)** |
| Model 2 ^b^ | 1 (reference) | **1.73 (1.28–2.34)** |
| Model 3 ^c^ | 1 (reference) | **1.68 (1.25–2.25)** |
| **HTP-only users** |  |  |
| Model 1 ^a^ | 1 (reference) | **1.77 (1.41–2.22)** |
| Model 2 ^b^ | 1 (reference) | **1.79 (1.42–2.24)** |
| Model 3 ^c^ | 1 (reference) | **1.79 (1.43–2.25)** |
| **Dual users** |  |  |
| Model 1 ^a^ | 1 (reference) | **2.53 (1.47–4.37)** |
| Model 2 ^b^ | 1 (reference) | **2.75 (1.50–5.04)** |
| Model 3 ^c^ | 1 (reference) | **2.71 (1.48–4.96)** |

Note: Odds ratios (ORs) and 95% confidence intervals (CIs) were estimated using logistic regression analyses. Missing outcome data were handled using multiple imputation, and 20 imputed datasets were generated. For multiple imputation, the number of previous smoking cessation attempts was modeled using collapsed categories (0, 1, 2, 3, and ≥4) to improve model stability.

Abbreviations: OR, odds ratio; CI, confidence interval; HTP, heated tobacco product.

^a^ Adjusted for age and sex.

^b^ Adjusted for age, sex, smoking duration, and number of previous smoking cessation attempts.

^c^ Adjusted for age, sex, smoking duration, number of previous smoking cessation attempts, and nicotine dependence.

In all models, companies were incorporated as clusters.

**Supplementary Table 6. Multiple imputation analysis of odds ratios for smoking cessation success according to combined categories of nicotine dependence and self-confidence for quitting among current smokers enrolled in a company-implemented smoking cessation program in Japan: a retrospective cohort study of employees from 38 companies conducted from May 2024 to March 2025.**

|  | Combination of nicotine dependence and self-confidence for quitting | | | |
| --- | --- | --- | --- | --- |
|  | High dependence and low self-confidence | Low dependence and  low self-confidence | High dependence and  high self-confidence | Low dependence and  high self-confidence |
| **Total participants** |  |  |  |  |
| Model 1 ^a^ | 1 (reference) | 1.10 (0.86–1.41) | **1.77 (1.50–2.10)** | **2.16 (1.56–3.00)** |
| Model 2 ^b^ | 1 (reference) | 1.09 (0.85–1.41) | **1.76 (1.51–2.07)** | **2.09 (1.51–2.90)** |
| **Cigarette-only smokers** |  |  |  |  |
| Model 1 ^a^ | 1 (reference) | 1.43 (0.91–2.27) | **1.60 (1.14–2.25)** | **2.71 (1.41–5.22)** |
| Model 2 ^b^ | 1 (reference) | 1.38 (0.89–2.15) | **1.60 (1.14–2.26)** | **2.66 (1.35–5.26)** |
| **HTP-only users** |  |  |  |  |
| Model 1 ^a^ | 1 (reference) | 0.92 (0.68–1.26) | **1.71 (1.40–2.10)** | **1.83 (1.23–2.72)** |
| Model 2 ^b^ | 1 (reference) | 0.91 (0.67–1.25) | **1.75 (1.41–2.16)** | **1.79 (1.19–2.69)** |
| **Dual users** |  |  |  |  |
| Model 1 ^a^ | 1 (reference) | 1.43 (0.65–3.17) | **2.68 (1.44–4.99)** | **2.76 (1.26–6.04)** |
| Model 2 ^b^ | 1 (reference) | 1.43 (0.62–3.30) | **2.92 (1.47–5.78)** | **3.03 (1.32–6.95)** |

Note: Odds ratios (ORs) and 95% confidence intervals (CIs) were estimated using logistic regression analyses. Missing outcome data were handled using multiple imputation, and 20 imputed datasets were generated. For multiple imputation, the number of previous smoking cessation attempts was modeled using collapsed categories (0, 1, 2, 3, and ≥4) to improve model stability.

Abbreviations: OR, odds ratio; CI, confidence interval; HTP, heated tobacco product.

^a^ Adjusted for age and sex.

^b^ Adjusted for age, sex, smoking duration, and number of previous smoking cessation attempts.

In all models, companies were incorporated as clusters.

**Supplementary Table 7. Sensitivity analysis of odds ratios for smoking cessation success according to nicotine dependence, assuming participants with missing outcome data were not successful in smoking cessation, among current smokers enrolled in a company-implemented smoking cessation program in Japan: a retrospective observational study of employees from 38 companies conducted from May 2024 to March 2025.**

|  | Nicotine dependence | |
| --- | --- | --- |
|  | High  (≤30 min after waking) | Low  (>30 min after waking) |
| **Total participants** | 1720 | 516 |
| Smoking cessation success, *n* (%) | 871  (50.6) | 281  (54.5) |
| Model 1 ^a^ | 1 (reference) | **1.21 (1.01–1.45)** |
| Model 2 ^b^ | 1 (reference) | 1.18 (0.98–1.42) |
| Model 3 ^c^ | 1 (reference) | 1.10 (0.92–1.31) |
| **Cigarette-only smokers** |  |  |
| Participants | 514 | 174 |
| Smoking cessation success, *n* (%) | 236  (45.9) | 96  (55.2) |
| Model 1 ^a^ | 1 (reference) | **1.61 (1.12–2.32)** |
| Model 2 ^b^ | 1 (reference) | **1.62 (1.11–2.36)** |
| Model 3 ^c^ | 1 (reference) | **1.54 (1.06–2.24)** |
| **HTP-only users** |  |  |
| Participants | 980 | 282 |
| Smoking cessation success, *n* (%) | 541  (55.2) | 155  (55) |
| Model 1 ^a^ | 1 (reference) | 1.02 (0.82–1.27) |
| Model 2 ^b^ | 1 (reference) | 1.00 (0.80–1.26) |
| Model 3 ^c^ | 1 (reference) | 0.92 (0.73–1.16) |
| **Dual users** |  |  |
| Participants | 226 | 60 |
| Smoking cessation success, *n* (%) | 94  (41.6) | 30  (50) |
| Model 1 ^a^ | 1 (reference) | 1.44 (0.77–2.68) |
| Model 2 ^b^ | 1 (reference) | 1.38 (0.73–2.63) |
| Model 3 ^c^ | 1 (reference) | 1.25 (0.69–2.24) |

Note: Odds ratios (ORs) and 95% confidence intervals (CIs) were estimated using logistic regression analyses.

Abbreviations: OR, odds ratio; CI, confidence interval; HTP, heated tobacco product.

^a^ Adjusted for age and sex.

^b^ Adjusted for age, sex, smoking duration, and number of previous smoking cessation attempts.

^c^ Adjusted for age, sex, smoking duration, number of previous smoking cessation attempts, and self-confidence for quitting.

In all models, companies were incorporated as clusters.

**Supplementary Table 8. Sensitivity analysis of odds ratios for smoking cessation success according to self-confidence for quitting, assuming participants with missing outcome data were not successful in smoking cessation, among current smokers enrolled in a company-implemented smoking cessation program in Japan: a retrospective observational study of employees from 38 companies conducted from May 2024 to March 2025.**

|  | Self-confidence for quitting | |
| --- | --- | --- |
|  | Low  (scores of 0−4) | High  (scores of 5−10) |
| **Total participants** | 1374 | 862 |
| Smoking cessation success, *n* (%) | 623  (45.3) | 529  (61.4) |
| Model 1 ^a^ | 1 (reference) | **1.84 (1.56–2.17)** |
| Model 2 ^b^ | 1 (reference) | **1.82 (1.56–2.13)** |
| Model 3 ^c^ | 1 (reference) | **1.81 (1.56–2.10)** |
| **Cigarette-only smokers** |  |  |
| Participants | 415 | 273 |
| Smoking cessation success, *n* (%) | 177  (42.7) | 155  (56.8) |
| Model 1 ^a^ | 1 (reference) | **1.73 (1.31–2.28)** |
| Model 2 ^b^ | 1 (reference) | **1.74 (1.33–2.28)** |
| Model 3 ^c^ | 1 (reference) | **1.69 (1.29–2.20)** |
| **HTP-only users** |  |  |
| Participants | 768 | 494 |
| Smoking cessation success, *n* (%) | 380  (49.5) | 316  (64) |
| Model 1 ^a^ | 1 (reference) | **1.74 (1.40–2.16)** |
| Model 2 ^b^ | 1 (reference) | **1.75 (1.42–2.17)** |
| Model 3 ^c^ | 1 (reference) | **1.77 (1.44–2.18)** |
| **Dual users** |  |  |
| Participants | 191 | 95 |
| Smoking cessation success, *n* (%) | 66  (34.6) | 58  (61.1) |
| Model 1 ^a^ | 1 (reference) | **2.68 (1.54–4.64)** |
| Model 2 ^b^ | 1 (reference) | **3.10 (1.64–5.84)** |
| Model 3 ^c^ | 1 (reference) | **3.05 (1.61–5.80)** |

Note: Odds ratios (ORs) and 95% confidence intervals (CIs) were estimated using logistic regression analyses.

Abbreviations: OR, odds ratio; CI, confidence interval; HTP, heated tobacco product.

^a^ Adjusted for age and sex.

^b^ Adjusted for age, sex, smoking duration, and number of previous smoking cessation attempts.

^c^ Adjusted for age, sex, smoking duration, number of previous smoking cessation attempts, and nicotine dependence.

In all models, companies were incorporated as clusters.

**Supplementary Table 9. Sensitivity analysis of odds ratios for smoking cessation success according to combined categories of nicotine dependence and self-confidence for quitting, assuming participants with missing outcome data were not successful in smoking cessation, among current smokers enrolled in a company-implemented smoking cessation program in Japan: a retrospective observational study of employees from 38 companies conducted from May 2024 to March 2025.**

|  | Combination of nicotine dependence and self-confidence for quitting | | | |
| --- | --- | --- | --- | --- |
|  | High dependence and low self-confidence | Low dependence and  low self-confidence | High dependence and  high self-confidence | Low dependence and  high self-confidence |
| **Total participants** | 1111 | 263 | 609 | 253 |
| Smoking cessation success, *n* (%) | 501  (45.1) | 122  (46.4) | 370  (60.8) | 159  (62.8) |
| Model 1 ^a^ | 1 (reference) | 1.08 (0.85–1.37) | **1.79 (1.52–2.10)** | **2.07 (1.49–2.87)** |
| Model 2 ^b^ | 1 (reference) | 1.08 (0.85–1.38) | **1.79 (1.55–2.08)** | **2.00 (1.46–2.75)** |
| **Cigarette-only smokers** |  |  |  |  |
| Participants | 324 | 91 | 190 | 83 |
| Smoking cessation success, *n* (%) | 133  (41.0) | 44  (48.4) | 103  (54.2) | 52  (62.7) |
| Model 1 ^a^ | 1 (reference) | 1.45 (0.96–2.20) | **1.62 (1.17–2.24)** | **2.64 (1.46–4.79)** |
| Model 2 ^b^ | 1 (reference) | 1.47 (0.97–2.25) | **1.64 (1.15–2.34)** | **2.69 (1.49–4.83)** |
| **HTP-only users** |  |  |  |  |
| Participants | 630 | 138 | 350 | 144 |
| Smoking cessation success, *n* (%) | 316  (50.2) | 64  (46.4) | 225  (64.3) | 91  (63.2) |
| Model 1 ^a^ | 1 (reference) | 0.89 (0.68–1.17) | **1.71 (1.42–2.06)** | **1.69 (1.13–2.54)** |
| Model 2 ^b^ | 1 (reference) | 0.89 (0.67–1.18) | **1.74 (1.43–2.12)** | **1.65 (1.10–2.49)** |
| **Dual users** |  |  |  |  |
| Participants | 157 | 34 | 69 | 26 |
| Smoking cessation success, *n* (%) | 52  (33.1) | 14  (41.2) | 42  (60.9) | 16  (61.5) |
| Model 1 ^a^ | 1 (reference) | 1.47 (0.66–3.31) | **2.83 (1.51–5.29)** | **3.00 (1.48–6.08)** |
| Model 2 ^b^ | 1 (reference) | 1.49 (0.63–3.55) | **3.40 (1.58–7.32)** | **3.23 (1.59–6.57)** |

Note: Odds ratios (ORs) and 95% confidence intervals (CIs) were estimated using logistic regression analyses.

Abbreviations: OR, odds ratio; CI, confidence interval; HTP, heated tobacco product.

^a^ Adjusted for age and sex.

^b^ Adjusted for age, sex, smoking duration, and number of previous smoking cessation attempts.

In all models, companies were incorporated as clusters.

**Supplementary Table 10. Baseline characteristics of current smokers enrolled in a company-implemented smoking cessation program in Japan under the assumption that participants with missing outcome data were not successful in smoking cessation, overall and stratified by smoking cessation status: a retrospective observational study of employees from 38 companies conducted from May 2024 to March 2025.**

|  |  | Total | | | | Cessation success | | | | Cessation failure | | | |  |
| --- | --- | --- | --- | --- | --- | --- | --- | --- | --- | --- | --- | --- | --- | --- |
|  |  | (n=2236) | | | | (n=1152) | | | | (n=1084) | | | | *P*-value |
| Age, years | | 46.2 | ± | 11 |  | 47.7 | ± | 10.4 |  | 44.5 | ± | 11.5 |  | <.001 |
| Sex, *n* (%) | |  |  |  |  |  |  |  |  |  |  |  |  | .74 |
|  | Male | 1986 | ( | 88.8 | ) | 1,027 | ( | 89.1 | ) | 959 | ( | 88.5 | ) |  |
|  | Female | 219 | ( | 9.8 | ) | 111 | ( | 9.6 | ) | 108 | ( | 10 | ) |  |
|  | Others | 31 | ( | 1.4 | ) | 14 | ( | 1.2 | ) | 17 | ( | 1.6 | ) |  |
| Type of Cigarettes Used, *n* (%) | |  |  |  |  |  |  |  |  |  |  |  |  | <.001 |
|  | Cigarette-only smokers | 688 | ( | 30.8 | ) | 332 | ( | 28.8 | ) | 356 | ( | 32.8 | ) |  |
|  | Heated tobacco product-only users | 1262 | ( | 56.4 | ) | 696 | ( | 60.4 | ) | 566 | ( | 52.2 | ) |  |
|  | Dual users | 286 | ( | 12.8 | ) | 124 | ( | 10.8 | ) | 162 | ( | 14.9 | ) |  |
| Smoking duration (years), *n* (%) | |  |  |  |  |  |  |  |  |  |  |  |  | .002 |
|  | 0–2 | 55 | ( | 2.5 | ) | 24 | ( | 2.1 | ) | 31 | ( | 2.9 | ) |  |
|  | 3–4 | 79 | ( | 3.5 | ) | 31 | ( | 2.7 | ) | 48 | ( | 4.4 | ) |  |
|  | 5–9 | 207 | ( | 9.3 | ) | 97 | ( | 8.4 | ) | 110 | ( | 10.1 | ) |  |
|  | 10–19 | 414 | ( | 18.5 | ) | 191 | ( | 16.6 | ) | 223 | ( | 20.6 | ) |  |
|  | 20–29 | 690 | ( | 30.9 | ) | 369 | ( | 32 | ) | 321 | ( | 29.6 | ) |  |
|  | 30+ | 791 | ( | 35.4 | ) | 440 | ( | 38.2 | ) | 351 | ( | 32.4 | ) |  |
| Cigarettes/day | | 17.8 | ± | 8.5 |  | 17.4 | ± | 8.5 |  | 18.1 | ± | 8.5 |  | .072 |
| Number of prior smoking cessation attempts, *n* (%) | |  |  |  |  |  |  |  |  |  |  |  |  | .67 |
|  | 0 | 698 | ( | 31.2 | ) | 361 | ( | 31.3 | ) | 337 | ( | 31.1 | ) |  |
|  | 1 | 672 | ( | 30.1 | ) | 347 | ( | 30.1 | ) | 325 | ( | 30 | ) |  |
|  | 2 | 365 | ( | 16.3 | ) | 197 | ( | 17.1 | ) | 168 | ( | 15.5 | ) |  |
|  | 3 | 266 | ( | 11.9 | ) | 135 | ( | 11.7 | ) | 131 | ( | 12.1 | ) |  |
|  | 4+ | 235 | ( | 10.5 | ) | 112 | ( | 9.7 | ) | 123 | ( | 11.3 | ) |  |
| Provision patterns of nicotine gums or patches, *n* (%) | |  |  |  |  |  |  |  |  |  |  |  |  | .30 |
|  | A: 7 sheets of 20-cm² patches + 96 pieces of gum | 1646 | ( | 73.6 | ) | 834 | ( | 72.4 | ) | 812 | ( | 74.9 | ) |  |
|  | B: 14 sheets of 10-cm² patches + 48 pieces of gum | 514 | ( | 23 | ) | 284 | ( | 24.7 | ) | 230 | ( | 21.2 | ) |  |
|  | C: 14 sheets of 20-cm² patches + 14 sheets of 10-mg patches | 35 | ( | 1.6 | ) | 15 | ( | 1.3 | ) | 20 | ( | 1.8 | ) |  |
|  | D: 20 sheets of 10-cm² patches | 9 | ( | 0.4 | ) | 4 | ( | 0.3 | ) | 5 | ( | 0.5 | ) |  |
|  | E: 96 pieces of gum alone | 32 | ( | 1.4 | ) | 15 | ( | 1.3 | ) | 17 | ( | 1.6 | ) |  |
| Importance of smoking cessation (score: 0–10) | | 6.9 | ± | 2.4 |  | 7.2 | ± | 2.3 |  | 6.7 | ± | 2.4 |  | <.001 |
| Nicotine dependence | |  |  |  |  |  |  |  |  |  |  |  |  | .13 |
|  | High (≤30 min after waking) | 1720 | ( | 76.9 | ) | 871 | ( | 75.6 | ) | 849 | ( | 78.3 | ) |  |
|  | Low (>30 min after waking) | 516 | ( | 23.1 | ) | 281 | ( | 24.4 | ) | 235 | ( | 21.7 | ) |  |
| Self-confidence for quitting | |  |  |  |  |  |  |  |  |  |  |  |  | <.001 |
|  | High (scores of 5−10) | 862 | ( | 38.6 | ) | 529 | ( | 45.9 | ) | 333 | ( | 30.7 | ) |  |
|  | Low (scores of 0−4) | 1374 | ( | 61.4 | ) | 623 | ( | 54.1 | ) | 751 | ( | 69.3 | ) |  |
| Posting frequency in the digital peer-supported app | | 31.4 | ± | 77 |  | 51.5 | ± | 98.7 |  | 10.1 | ± | 31.8 |  | <.001 |
| Posting approvals from group members | | 52.7 | ± | 99 |  | 88.1 | ± | 121.2 |  | 14.9 | ± | 43.2 |  | <.001 |

Note*:* Data are presented as means and standard deviations (SDs) for continuous variables and as numbers (percentages) for categorical variables. The Pearson chi-squared test was used for categorical variables and the Mann–Whitney U test was used for continuous variables, as appropriate.

**Supplementary Table 11.** Baseline characteristics of current smokers enrolled in a company-implemented smoking cessation program in Japan, according to inclusion in the analytic sample or exclusion due to missing outcome data: a retrospective observational study of employees from 38 companies conducted from May 2024 to March 2025.

|  |  | Analysis sample | | | | Missing outcome | | | |  |
| --- | --- | --- | --- | --- | --- | --- | --- | --- | --- | --- |
|  |  | (n=2143) | | | | (n=93) | | | | *P*-value |
| Age, years | | 46.5 | ± | 10.9 |  | 39.2 | ± | 12 |  | <.001 |
| Sex, *n* (%) | |  |  |  |  |  |  |  |  | .49 |
|  | Male | 1903 | ( | 88.8 | ) | 83 | ( | 89.2 | ) |  |
|  | Female | 209 | ( | 9.8 | ) | 10 | ( | 10.8 | ) |  |
|  | Others | 31 | ( | 1.4 | ) | 0 | ( | 0 | ) |  |
| Type of Cigarettes Used, *n* (%) | |  |  |  |  |  |  |  |  | .004 |
|  | Cigarette-only smokers | 659 | ( | 30.8 | ) | 29 | ( | 31.2 | ) |  |
|  | Heated tobacco product-only users | 1220 | ( | 56.9 | ) | 42 | ( | 45.2 | ) |  |
|  | Dual users | 264 | ( | 12.3 | ) | 22 | ( | 23.7 | ) |  |
| Smoking duration (years), *n* (%) | |  |  |  |  |  |  |  |  | <.001 |
|  | 0–2 | 51 | ( | 2.4 | ) | 4 | ( | 4.3 | ) |  |
|  | 3–4 | 68 | ( | 3.2 | ) | 11 | ( | 11.8 | ) |  |
|  | 5–9 | 190 | ( | 8.9 | ) | 17 | ( | 18.3 | ) |  |
|  | 10–19 | 391 | ( | 18.2 | ) | 23 | ( | 24.7 | ) |  |
|  | 20–29 | 673 | ( | 31.4 | ) | 17 | ( | 18.3 | ) |  |
|  | 30+ | 770 | ( | 35.9 | ) | 21 | ( | 22.6 | ) |  |
| Cigarettes/day | | 17.8 | ± | 8.4 |  | 17.6 | ± | 10.9 |  | .90 |
| Number of prior smoking cessation attempts, *n* (%) | |  |  |  |  |  |  |  |  | .24 |
|  | 0 | 666 | ( | 31.1 | ) | 32 | ( | 34.4 | ) |  |
|  | 1 | 645 | ( | 30.1 | ) | 27 | ( | 29 | ) |  |
|  | 2 | 355 | ( | 16.6 | ) | 10 | ( | 10.8 | ) |  |
|  | 3 | 257 | ( | 12.0 | ) | 9 | ( | 9.7 | ) |  |
|  | 4+ | 220 | ( | 10.3 | ) | 15 | ( | 16.1 | ) |  |
| Provision patterns of nicotine gums or patch, *n* (%) | |  |  |  |  |  |  |  |  | .057 |
|  | A: 7 sheets of 20-cm² patches + 96 pieces of gum | 1584 | ( | 73.9 | ) | 62 | ( | 66.7 | ) |  |
|  | B: 14 sheets of 10-cm² patches + 48 pieces of gum | 491 | ( | 22.9 | ) | 23 | ( | 24.7 | ) |  |
|  | C: 14 sheets of 20-cm² patches + 14 sheets of 10-mg patches | 32 | ( | 1.5 | ) | 3 | ( | 3.2 | ) |  |
|  | D: 20 sheets of 10-cm² patches | 8 | ( | 0.4 | ) | 1 | ( | 1.1 | ) |  |
|  | E: 96 pieces of gum alone | 28 | ( | 1.3 | ) | 4 | ( | 4.3 | ) |  |
| Importance of smoking cessation (score: 0–10) | | 7 | ± | 2.4 |  | 6.6 | ± | 2.6 |  | .12 |
| Nicotine dependence | |  |  |  |  |  |  |  |  | .25 |
|  | High (≤30 min after waking) | 1653 | ( | 77.1 | ) | 6.7 | ( | 72 | ) |  |
|  | Low (>30 min after waking) | 490 | ( | 22.9 | ) | 26 | ( | 28 | ) |  |
| Self-confidence for quitting | |  |  |  |  |  |  |  |  | .087 |
|  | High (scores of 5−10) | 834 | ( | 38.9 | ) | 28 | ( | 30.1 | ) |  |
|  | Low (scores of 0−4) | 1309 | ( | 61.1 | ) | 65 | ( | 69.9 | ) |  |
| Posting frequency in the digital peer-supported app | | 32.6 | ± | 78.4 |  | 3.1 | ± | 7.6 |  | <.001 |
| Posting approvals from group members | | 54.8 | ± | 100.6 |  | 3.5 | ± | 10.5 |  | <.001 |

*Note:* Data are presented as means and standard deviations (SDs) for continuous variables and as numbers (percentages) for categorical variables. The Pearson chi-square test was used for categorical variables, as appropriate. The Mann–Whitney U test was used for continuous variables.

**Supplementary Figure 1.** Conceptual framework of the associations between nicotine dependence, self-confidence for quitting, and smoking cessation.

**
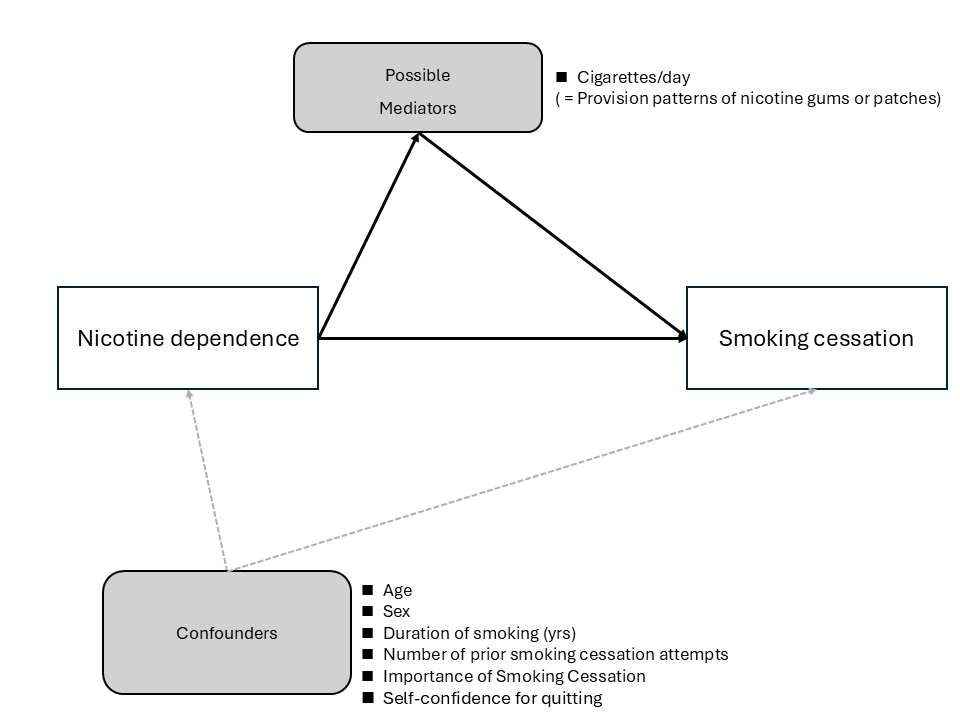
**

**Panel A.** Association between nicotine dependence and smoking cessation success rate. *Note:* *Daily cigarette consumption* may mediate the pathway from nicotine dependence to successful smoking cessation. Specifically, stronger nicotine dependence tends to lead to higher cigarette consumption, which, in turn, influences the provision of more intensive nicotine replacement therapy (e.g., higher-dose patches or combined use of gum) and ultimately affects cessation outcomes.

**
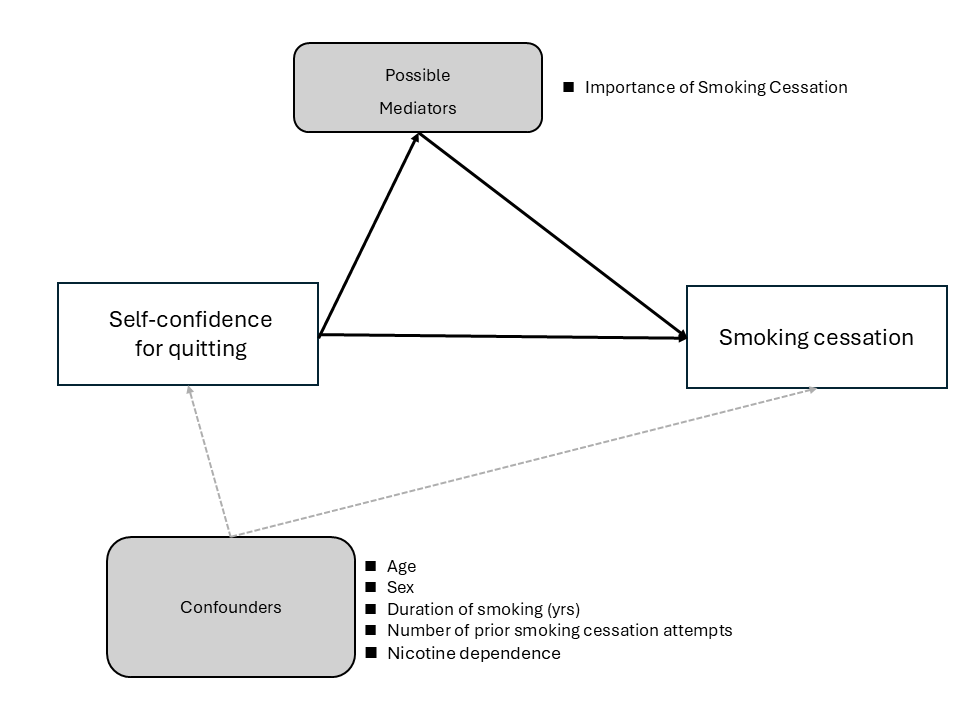
**

**Panel B.** Association between self-confidence for quitting and cessation success rate. *Note:* Higher self-efficacy may improve the *perceived importance* of quitting and form a psychological mediation pathway.

**
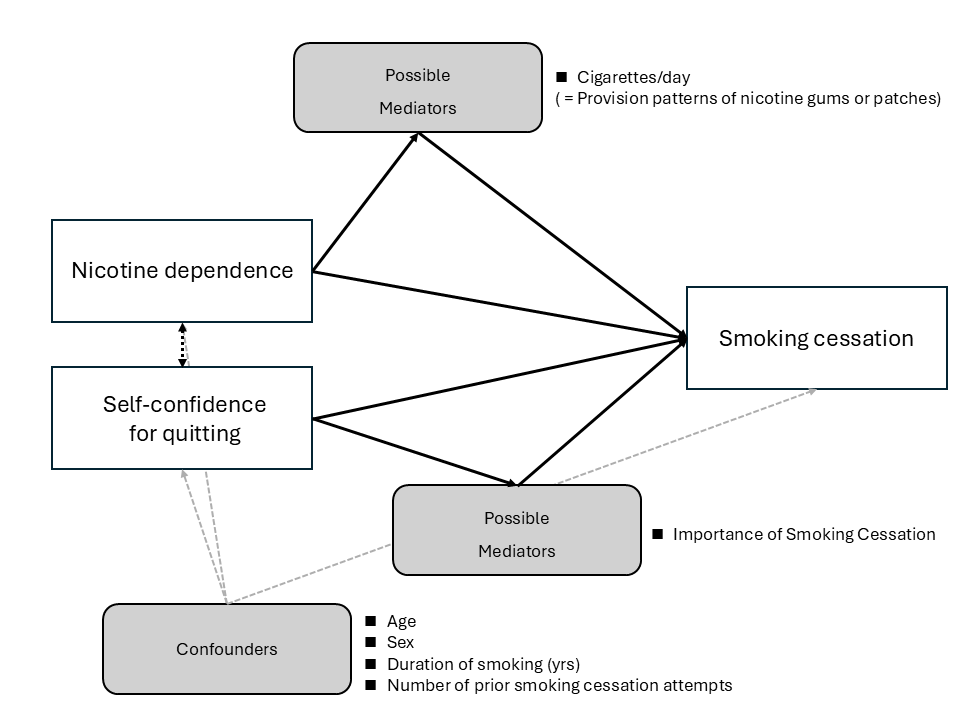
**

**Panel C.** Combined association of nicotine dependence and self-confidence for quitting with cessation success. ***Note:*** *Nicotine dependence* and self-confidence for quitting were correlated, but were not assumed to have a direct causal relationship. These potential mediators were not adjusted for in the main analysis to avoid over-adjustment and preserve the total effect.
